# Cathode Catalyst Layer Design in PEM Water Electrolysis toward Reduced Pt Loading and Hydrogen Crossover

**DOI:** 10.1021/acsami.4c01827

**Published:** 2024-04-23

**Authors:** Zheyu Zhang, Axelle Baudy, Andrea Testino, Lorenz Gubler

**Affiliations:** † Electrochemistry Laboratory, Paul Scherrer Institut, 5232 Villigen PSI, Switzerland; ‡ Laboratory for Sustainable Energy Carriers and Processes, Paul Scherrer Institut, 5232 Villigen PSI, Switzerland; § STI SMX-GE, École Polytechnique Fédérale de Lausanne, 1015 Lausanne, Switzerland

**Keywords:** PEM water electrolysis, hydrogen crossover, cathode catalyst layer, Pt loading, Pt/C electrocatalyst, carbon support, ionomer content

## Abstract

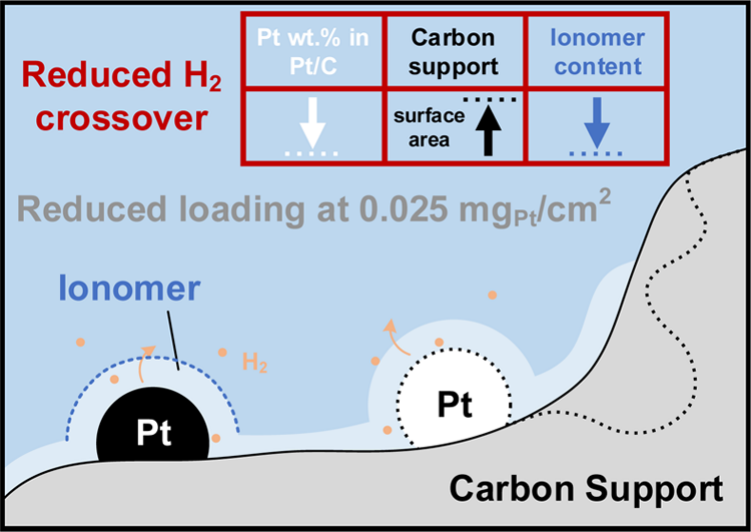

Reducing the use of platinum group metals is crucial for the large-scale deployment of proton exchange membrane (PEM) water electrolysis systems. The optimization of the cathode catalyst layer and decrease of the cathode Pt loading are usually overlooked due to the predominant focus of research on the anode. However, given the close relationship between the rate of hydrogen permeation through the membrane in an operating cell and the local hydrogen concentration near the membrane–cathode interface, the structural design of the cathode catalyst layer is considered to be of pivotal importance for reducing H_2_ crossover, particularly in combination with the use of thin (≲50 μm) membranes. In this study, we have conducted a detailed investigation on the cathode structural parameters, covering the Pt wt % of the Pt/C electrocatalyst, the type of carbon support (Vulcan and high surface area carbon, HSAC), and the ionomer content, with a goal to reduce Pt loading to 0.025 mg_Pt_/cm^2^ while minimizing the rate of cell hydrogen crossover. We found that the electrochemical performance is mainly influenced by the changes in the interfacial contact resistance due to variations in the cathode thickness. Both the Pt wt % in Pt/C and the ionomer content showed a positive correlation with the measured H_2_ in O_2_% in the anode outlet, whereas the Pt loading exhibited an opposite trend. The rate of hydrogen crossover was analyzed in relation to the calculated local volumetric current density within the cathode catalyst layer. Based on the obtained hydrogen mass transfer coefficient, a cathode catalyst layer comprising 40 wt % Pt on HSAC support with an ionomer-to-carbon (I/C) ratio of 0.35 was found to be an optimum configuration for achieving a low Pt loading of 0.025 mg_Pt_/cm^2^ and a reduced rate of hydrogen crossover.

## Introduction

Proton exchange membrane (PEM) water electrolysis is a key technology for the Energy Transition. It produces high-purity hydrogen gas suitable for fuel cell-based mobility, industrial processes, and seasonal storage. Platinum group metals (PGMs) are conventionally used as catalysts for electrode reactions due to their outstanding catalytic activity and chemical stability in harsh acidic and oxidizing conditions.^1^ On the cathode side, commercial carbon-supported platinum (Pt/C) remains a state-of-the-art choice for the hydrogen evolution reaction (HER). While a high Pt loading between 0.5 and 1.0 mg_Pt_/cm^2^ is commonly used,^2,3^ a reduction of the Pt loading to 0.05–0.2 mg_Pt_/cm^2^, or further below 0.05 mg_Pt_/cm^2^, is desired for reducing the cost of PGM usage in megawatt-scale and larger PEM water electrolysis systems in the long term.^2,4−6^

Given the fast kinetics of the HER in acidic medium, studies have shown that the amount of Pt can be decreased considerably. Recent publications have reported Pt loadings of 0.1 mg_Pt_/cm^2^ or less.^7−9^ Bernt et al. performed a comparison of the cathode Pt loading of 0.025 and 0.3 mg_Pt_/cm^2^ and reported similar beginning-of-life cell performance.^10^ The work is one of the few studies in the literature that has looked into an ultralow Pt loading at a level of 0.025 mg_Pt_/cm^2^. Considering the compositional complexity of the catalyst layer, requiring a dedicated balance of the electronic, ionic, and void phases with distributed catalytic active sites, a systematic and parametric cathode catalyst layer design investigation on the impact of Pt loading reduction is needed.

Alongside an interest in assessing the effect of low-Pt cathodes on the electrochemical performance of the cell, we are also focused on evaluating the hydrogen crossover characteristics, which are anticipated to differ based on the cathode design parameters. The catalyst layer structural parameters usually include the type of carbon support, the Pt weight percentage (Pt wt %) on the carbon support, and the ionomer content, characterized by the ionomer-to-carbon (I/C) ratio. In a working PEM water electrolysis cell, hydrogen crossover occurs from the cathode to the anode and is driven mainly by a concentration gradient of the dissolved hydrogen in an aqueous state.^11−13^ The crossover hydrogen lowers the faradaic efficiency and poses safety risks by forming a potentially explosive gas mixture with oxygen in the anode chamber. To quantify the rate of permeation, the concentration of hydrogen at the source of diffusion can be calculated, in an ideal case, using Henry’s law by assuming an equilibrium between the liquid and gas phases. It has been, however, argued that the experimentally measured crossover rate in an operating cell could suggest a higher source concentration than the saturation level due to structure-related mass transfer limitations in the cathode catalyst layer. Trinke et al. demonstrated that the crossover rate is dependent on the current density of the electrolysis cell, exhibiting an increasing trend with an increased current density.^14^ The reason has been found to be predominantly a supersaturated hydrogen concentration near the catalyst particle within the aqueous domain of the ionomer film.^14^ Subsequently, it was discussed that the cathode I/C ratio has a large influence on the rate of hydrogen permeation.^15,16^ For example, a higher I/C ratio yields a thicker ionomer film, which extends the length of the diffusion pathways required for the produced dissolved hydrogen to reach the pore space. This results in a more pronounced local hydrogen concentration and an increased crossover rate.^15^ Other factors such as cell compression, which causes microstructure changes involving the catalyst layer ionomer-void surface area, were found to affect the effectiveness of the gas mass transport and therefore the rate of hydrogen crossover.^17^ All in all, it can be inferred that an appropriate cathode structural design is crucial for optimizing the characteristics governing the hydrogen permeation processes in an electrolysis cell. Nevertheless, to date, relevant reports are still limited in this matter, especially with low Pt loadings, and the understanding is far from complete.

**Scheme 1:**
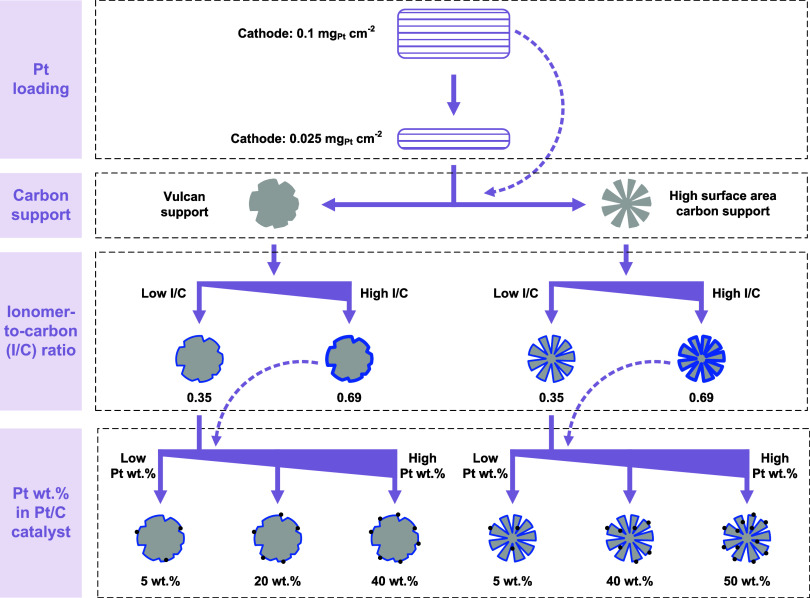
An Overview Illustrating the Variations in Structural Parameters and the Corresponding Cathode Catalyst Layer Configurations Studied Different Pt loadings, carbon support types, ionomer–to-carbon ratios, and Pt wt % in Pt/C catalysts were surveyed, via the use of various commercial Pt/C catalysts, modifications in the amount of ionomer during ink preparation, and the adjustments in the target spraying loading during spray coating. The dashed line indicates alternative combinations of the structural parameters included in this work.

In the current work, we present a detailed study aiming to concurrently decrease the loading of Pt and minimize the rate of hydrogen crossover through the analysis of cathode catalyst layers featuring a variety of structural parameter combinations. A 4-fold reduction of Pt was achieved from 0.1 to 0.025 mg_Pt_/cm^2^. The Pt/C catalyst was chosen from two types of carbon support, Vulcan carbon and high surface area carbon (HSAC), and from a range of Pt wt % in Pt/C between nominally 5 and 50 wt %. Two levels of the ionomer content, represented by I/C mass ratios of 0.35 and 0.69, were employed. An overview of the different cathode catalyst layer compositions investigated in this study is given in Scheme 1. Electrolysis cell measurements were carried out, and the electrochemical performance of the cell containing the various cathode configurations was compared. In addition, we aimed to clarify the connection between the cell hydrogen crossover and the cathode structure by estimating the local hydrogen concentration profile within the catalyst layer through the interpretation of the obtained results. We demonstrate that a reduction of Pt loading could cause additional ohmic losses due to increased contact resistance. A decrease of Pt wt % in Pt/C, I/C ratio, or an increase of the Pt loading was proven to be typically beneficial in decreasing the rate of hydrogen permeation. At a reduced Pt loading of 0.025 mg_Pt_/cm^2^, the cathode of 40 wt % Pt on HSAC support with an I/C ratio of 0.35 shows the highest calculated hydrogen mass transfer coefficient, which is seen as the optimal configuration. These findings help to provide guidance for the design criteria of next-generation PEM water electrolysis cells.

## Results and Discussion

### Microstructure of the Cathode Catalyst Layer

**Figure 1:**
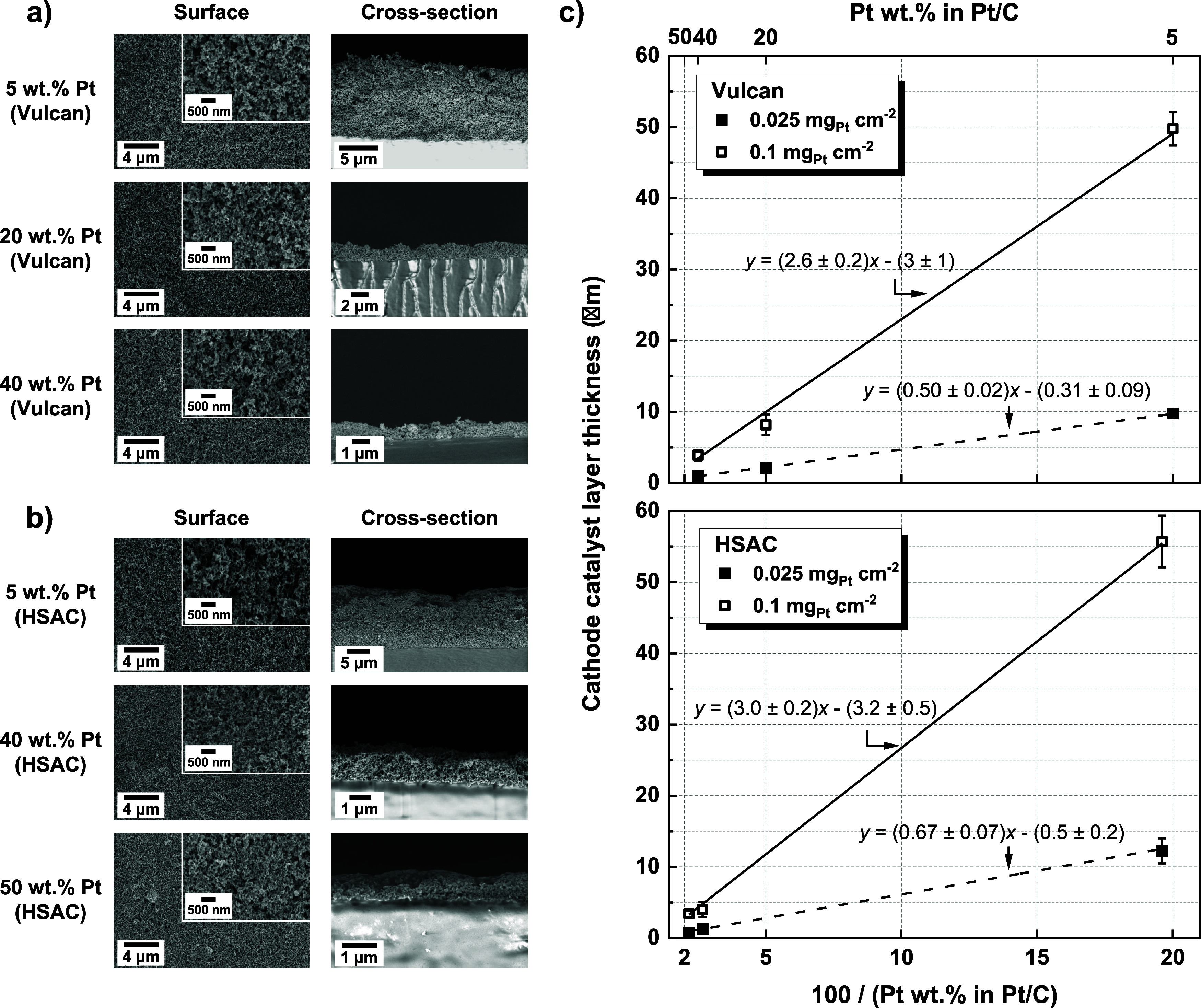
SEM surface and cross-sectional images for cathode catalyst layers (0.025 mg_Pt_/cm^2^ and an I/C ratio of 0.35) with (a) Vulcan carbon-supported Pt/C catalysts and (b) HSAC-supported Pt/C catalysts. The thickness of the cathode catalyst layers (0.025 and 0.1 mg_Pt_/cm^2^ and an I/C ratio of 0.35) is plotted in (c) as a function of the inverse Pt wt % in Pt/C, where the actual instead of nominal (cf. Experimental Section) Pt wt % values are used for HSAC-supported Pt/C catalysts.

The preparation of the cathode catalyst layer is described in the Experimental Section. Generally, all spray-coated cathode catalyst layers show a uniform Pt/C particle distribution on the membrane surface. Examples of top-view and cross-sectional images taken by SEM, with an I/C ratio of 0.35 and a Pt loading of 0.025 mg_Pt_/cm^2^ for the selected samples, are demonstrated in Figure 1a,b. An increase in Pt wt % in Pt/C, at a fixed Pt loading, leads to a decrease in the catalyst layer thickness. In the case of a relatively high Pt wt %, such as 50 wt % with HSAC, the thickness is in the range of ∼1 μm. Nonetheless, a continuous surface and cross-sectional morphology is still observed. Additional SEM images for samples with an I/C ratio of 0.35 and a Pt loading of 0.1 mg_Pt_/cm^2^ are displayed in Figure S1 (Vulcan carbon) and Figure S2 (HSAC). On the other hand, an increase in the ionomer content, from an I/C ratio of 0.35 to 0.69, is reported not to affect the catalyst layer thickness, as the ionomer is only expected to fill up the existing pore space within the studied I/C ratio range.^18^ The surface morphology is observed to show minimal difference, as evidenced in Figure S3 using 5 wt % Pt on Vulcan carbon with a Pt loading of 0.025 mg_Pt_/cm^2^.

The measured catalyst layer thickness is therefore summarized in Figure 1c, for all samples with an I/C ratio of 0.35. A linear trend is observed between the thickness and the reciprocal of the Pt mass fraction in Pt/C (100/Pt wt % in Pt/C). This is due to the fact that for Vulcan and Ketjen-black supported Pt/C catalyst, it has been reported that the electrode thickness is only linearly dependent on the carbon loading when the I/C ratio is ≤3 (eq 1, where δ_CL_ is the catalyst layer thickness in μm and *L*_C_ is the carbon loading in mg/cm^2^):^19,20^
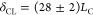
1

Since at a given Pt loading the quantity of carbon in a Pt/C catalyst is directly related to the reciprocal of the Pt, the mass relationship thus translates into a linear dependency as shown in eq 2, where *L*_Pt_ is the Pt loading in mg/cm^2^:
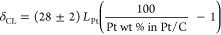
2

The absolute values of both the slope and the intercept in eq 2 are (28 ± 2)·*L*_Pt_. For Pt loadings of 0.025 and 0.1 mg_Pt_/cm^2^, the absolute values therefore correspond to 0.70 ± 0.05 and 2.8 ± 0.2, respectively. As shown in Figure 1c, in general, the slopes and intercepts fitted via regression using the experimentally measured catalyst layer thickness can approximate the calculated values from the literature within the error bar range.

### Electrochemical Performance

**Figure 2:**
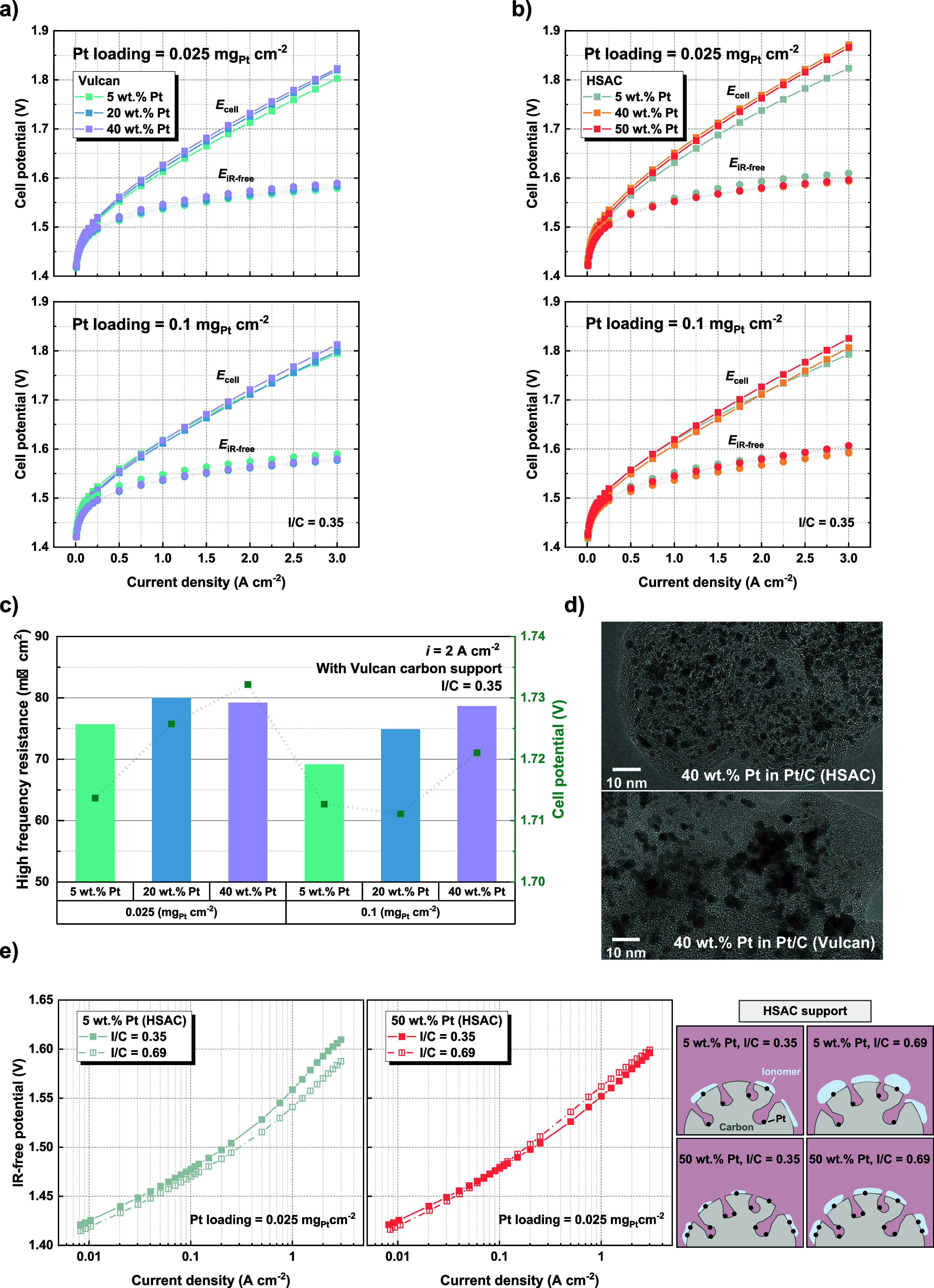
Polarization curves and *iR*-free potentials at Pt loadings of 0.025 and 0.1 mg_Pt_/cm^2^ (I/C ratio = 0.35) for (a) Vulcan carbon-supported Pt/C catalysts and (b) HSAC-supported Pt/C catalysts. (c) A summary of the high-frequency resistance and cell potential at 2 A/cm^2^ (I/C ratio = 0.35) for Vulcan carbon-supported Pt/C catalysts. (d) TEM images of 40 wt % Pt in Pt/C catalysts on HSAC and Vulcan carbon supports. (e) *IR*-free potentials for 5 and 50 wt % Pt in Pt/C catalysts on HSAC support at a Pt loading of 0.025 mg_Pt_/cm^2^; the schematic diagrams demonstrate the ionomer coverage on the carbon support depending on the I/C ratio. Both the cathode and anode pressures are maintained under ambient conditions.

Electrolysis cell measurements were conducted using catalyst-coated membranes (CCMs), by varying the cathode catalyst layer while keeping all of the other components identical. A reduction in Pt loading from 0.1 to 0.025 mg/cm^2^ at an I/C ratio of 0.35 demonstrates a minor impact on the cell’s polarization curve, as illustrated in Figure 2a,b. This change is primarily ascribed to an increase in the high-frequency resistance (HFR). Taking Vulcan carbon electrodes as an example, the HFR increase when reducing Pt loading from 0.1 to 0.025 mg_Pt_/cm^2^ is observed to be more pronounced for samples with a lower Pt wt % in Pt/C than for those with a higher Pt wt % (Figure 2c). For instance, the measured increase in HFR is 7 mΩ·cm^2^ at 2 A/cm^2^ for 5 wt % Pt in Pt/C, while the respective value is 1 mΩ·cm^2^ for 40 wt % Pt. The increase in HFR is believed to be due to increased contact resistance, resulting from a reduced thickness of the cathode catalyst layer with a decreased Pt loading. The relatively greater HFR increase at a lower Pt wt % is likely because of a more significant absolute change in the cathode catalyst layer thickness than for a higher Pt wt %. As evidenced in Figure 1c, the decrease of the catalyst layer thickness upon reducing the Pt loading is from 40 μm for 5 wt % Pt to 3 μm for 40 wt % Pt for Vulcan carbon electrodes. The HFR, in Figure 2c, mostly reflects the relative trend of the cell potential across different cathode catalyst layers. However, it is also worth noting that the HFR differences measured here are close to the CCM sample-to-sample variation, which could also arise from the variations during cell assembly. The corresponding HFR and cell potential comparison for HSAC electrodes is shown in Figure S4.

To further assess the effect of the different cathode configurations, the contribution of the ohmic overpotential is subtracted from the cell potential. The resulting *iR*-free potential is then plotted against the current density, as depicted in Figure 2a,b. All CCMs display an HFR-corrected cell potential varying within a range of 32 mV between 1.561 and 1.593 V at 2 A/cm^2^. The *iR*-free potential typically includes the reversible potential, kinetic overpotential, and mass transport overpotential. With a constant reversible potential, the kinetic overpotential usually dominates over the mass transport overpotential in the low current density region. In this region, the observed similarity of *iR*-free potentials, particularly for the two different Pt loadings, suggests rapid cathode HER kinetics – a conclusion well-supported by the findings in the literature.^10^ A comparison of the influence of the ionomer content is illustrated in Figure 2e. It can be seen that doubling the amount of ionomer from an I/C ratio of 0.35 to 0.69 does not substantially affect the *iR*-free potential in the kinetic domain for HSAC electrodes with 5 and 50 wt % Pt in Pt/C. The consistent observations may indicate that the aforementioned variations in cathode catalyst layer parameters are likely to produce only slight effects, which are largely overwhelmed by the much more pronounced overpotential of the oxygen evolution reaction (OER) at the anode.

At higher current densities, however, in Figure 2e, the electrode with an I/C ratio of 0.35 and 5 wt % Pt in Pt/C exhibits a moderately increased mass transport overpotential compared to the other samples. The observation is counterintuitive since lower ionomer content usually reduces bubble accumulation at the catalyst layer, thereby reducing the mass transport resistance. One possible reason for this could be a lower proton conductivity of the cathode catalyst layer because of a low ionomer content, combined with the effects of a low Pt wt %, a porous carbon substrate, and a reduced roughness factor due to a low Pt loading of 0.025 mg_Pt_/cm^2^. Typically, the proton conductivity within the catalyst layer increases as the I/C ratio increases,^21^ which explains the lack of such an issue in cathodes with an I/C ratio of 0.69 in Figure 2e. Additionally, the Pt particles are considered largely to be situated within the pores and sparsely distributed for the 5 wt % Pt catalyst.^22^ In contrast, with a higher Pt wt %, there is a denser distribution of the Pt particles, of which a larger portion is found on the exterior of the carbon support.^22^ Therefore, with a limited ionomer content (i.e., an I/C ratio of 0.35), the cathode with 50 wt % Pt could allow more efficient proton transport to a greater amount of Pt active sites, yielding a reduced mass transport resistance compared to the cathode with 5 wt % Pt. Moreover, the electrochemical surface area (ECSA) of the electrode, using a symmetrical CCM with both sides employing the same 5 wt % Pt in Pt/C (an I/C ratio of 0.35) catalyst layer, is measured to be 136 m^2^/g_Pt_ (Figure S5). Accounting for a Pt loading of 0.025 mg_Pt_/cm^2^, it corresponds to an electrode roughness factor of 34. At high geometric current densities, the relatively low cathode roughness factor may contribute to the observed mass transport limitations. Finally, in comparison to Vulcan carbon, HSAC used in Figure 2e has a higher surface area and an increased pore volume, leading to less continuous proton transport pathways and therefore a lower proton conductivity within the catalyst layer.^21,23^ This is illustrated in Figure S6, where the cathode (5 wt % Pt and an I/C ratio of 0.35) with Vulcan carbon support shows less mass transport limitations than the one with HSAC shown in Figure 2e.

Figure 2d presents TEM images showing microstructures of HSAC and Vulcan carbon, both containing 40 wt % Pt in Pt/C. The Pt particles on HSAC are slightly smaller than those on Vulcan. This observation aligns with the research conducted by Sneed et al., who reported Pt particle sizes of approximately 3 nm for HSAC and 4 nm for Vulcan at the identical Pt weight percentage.^22^ In their study, it is indicated that as the Pt wt % increases from 5 to 40 wt %, a consistent Pt particle size of around 3 nm can be maintained on HSAC, attributed to its greater available surface area and ability to confine Pt particles within the pores.^22^ Conversely, the Pt particle size on Vulcan carbon is noted to gradually increase from around 2.3 to 4 nm over the same range of Pt weight percentage.^22^

### Rate of Hydrogen Crossover

Experimental measurement of hydrogen crossover in an operating cell is conducted by analyzing the content of H_2_ in the anode O_2_ product stream (H_2_ in O_2_%), across a range of current densities between 0.2 and 3 A/cm^2^. Repeats of selected measurements were conducted and are shown in Figures S7–S9. The sample standard deviation relative to the mean is calculated to be between 2 and 8%. Consequently, we expect the measurement uncertainty to fall within a similar range, and the difference we observed between various cathode configurations is still significant.

Typically, and as confirmed by most of our observations, the H_2_ in the O_2_% tends to decrease with increasing current density. This decrease is attributed to an increased O_2_ flux generated at the anode, which effectively dilutes the hydrogen crossing over from the cathode. In certain cases, it is reported that the H_2_ in O_2_% begins to increase again at high current densities due to additional influential factors.^24,25^ In our experiments, three different cathode pressure levels were employed: ambient (nominally 1 bar), 3 bar, and 7 bar. The anode chamber is not pressurized and was maintained at ambient level. As the crossover hydrogen flux is predominately driven by a diffusion mechanism, it generally increases when the cathode pressure is increased.

**Figure 3:**
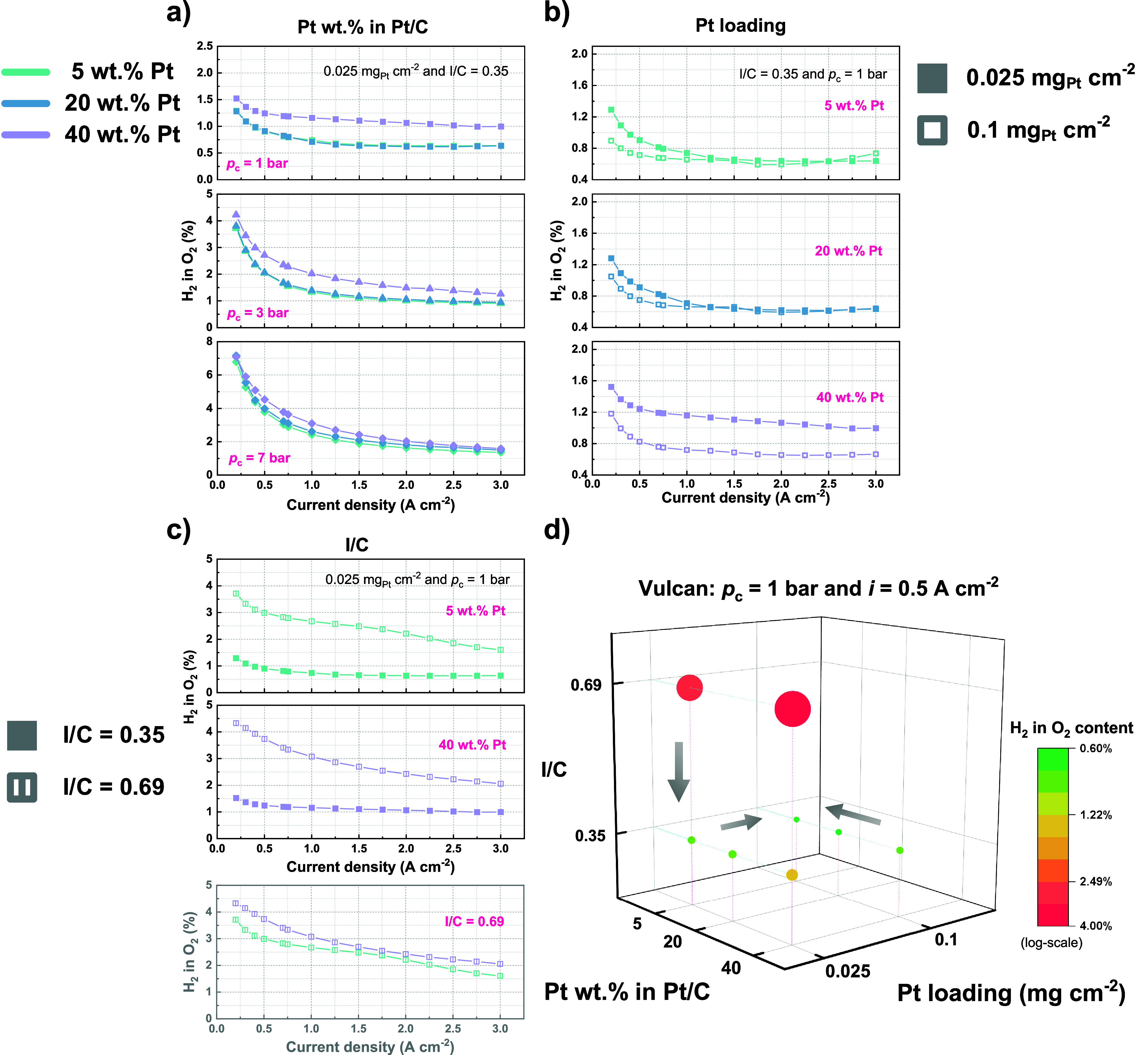
Effect of (a) Pt wt % in Pt/C, (b) Pt loading, and (c) I/C ratio on H_2_ in O_2_% using Vulcan carbon-supported Pt/C catalysts. A summary of the measured H_2_ in O_2_% at 0.5 A/cm^2^ is shown in (d). Ambient pressure at the anode is maintained for all measurements.

Figure 3 compares the H_2_ in O_2_% of cells with various cathode configurations, examining the impact of Pt content in wt % in Pt/C (Figure 3a), Pt loading (Figure 3b), and ionomer content (Figure 3c), all using Vulcan carbon as the catalyst support. At lower current densities, the H_2_ in O_2_%, as previously noted, is typically higher. It functions as a key factor in evaluating safety risks associated with the potential formation of explosive H_2_/O_2_ mixtures, thereby determining the safe operational boundaries of the electrolysis cell. On the other hand, as the net crossover hydrogen flux could increase with increasing current density, results obtained at higher current densities are more indicative of the upper limit of the cell’s Faraday efficiency losses due to hydrogen crossover.

With a fixed Pt loading of 0.025 mg_Pt_/cm^2^ and an I/C ratio of 0.35, Figure 3a illustrates that an increase in Pt wt % generally leads to a higher H_2_ in O_2_%, a trend observed consistently across all three cathode pressure levels. As depicted in Figure 3b, increasing the Pt loading from 0.025 to 0.1 mg_Pt_/cm^2^, while keeping the other cathode structural parameters constant, results in a decrease in H_2_ in O_2_%, for each of the three cells using different Pt wt % in Pt/C catalysts. Furthermore, as demonstrated in Figure 3c, increasing the ionomer content by doubling the I/C ratio from 0.35 to 0.69 notably increases the H_2_ in O_2_%, for example, causing an approximately 3-fold increase at a current density of 0.5 A/cm^2^. At a higher I/C ratio of 0.69, as evidenced in the bottom graph of Figure 3c, the comparison of Pt wt % supports the findings from Figure 3a: the measurement using 40 wt % Pt catalyst exhibits a higher H_2_ in O_2_% than that using 5 wt % Pt.

Figure 3d displays a comparative overview of the measured H_2_ in O_2_% at 0.5 A/cm^2^. It becomes clear that to achieve minimal hydrogen crossover, a cathode configuration with a lower Pt wt % in Pt/C, a lower I/C ratio, and preferably a higher Pt loading is of advantage. Particularly, at a reduced Pt loading of 0.025 mg_Pt_/cm^2^, employing a combination of 5 wt % Pt in Pt/C and an I/C ratio of 0.35 appears to be the most effective structure for this purpose.

**Figure 4:**
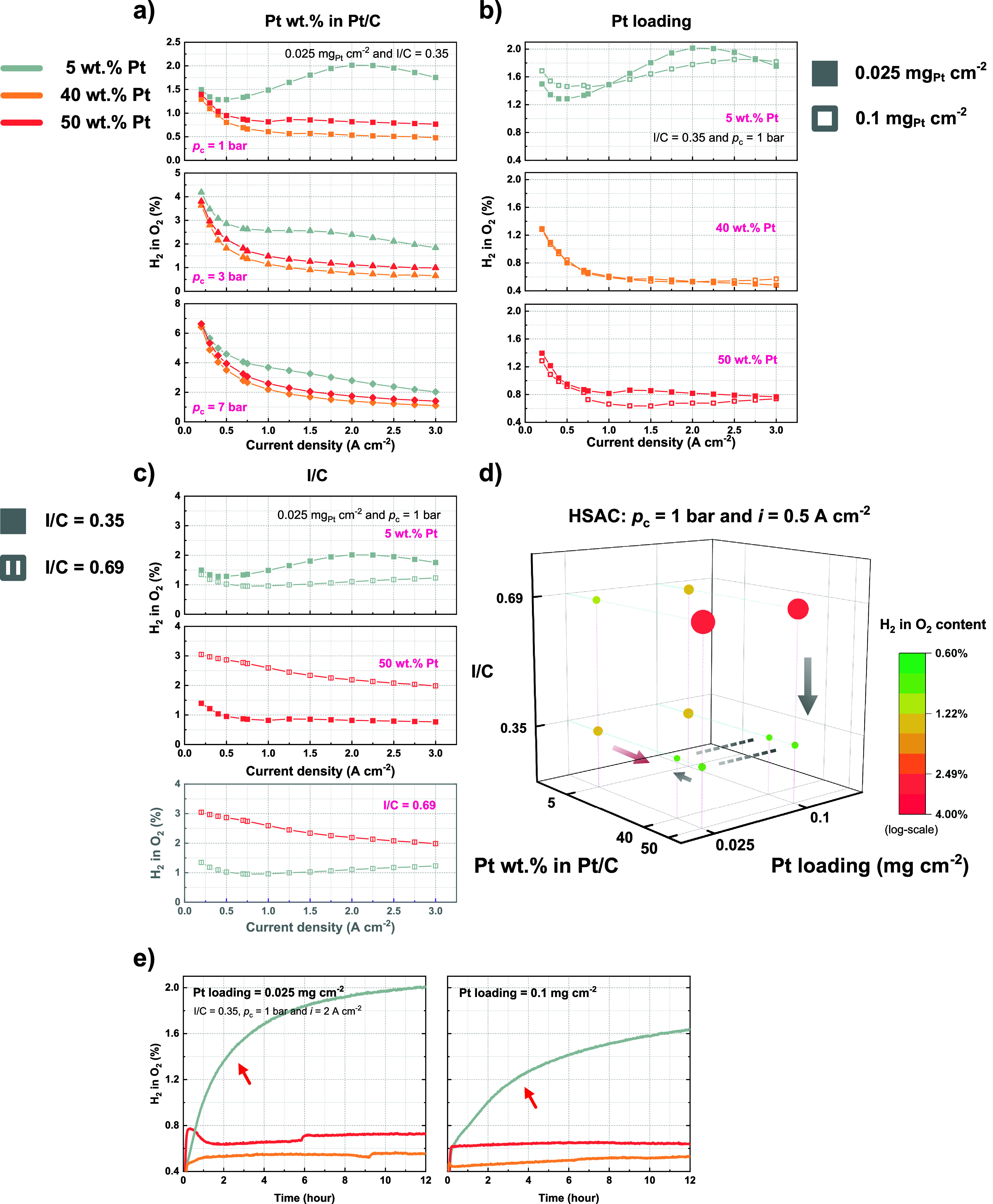
Effect of (a) Pt wt % in Pt/C, (b) Pt loading, and (c) I/C ratio on H_2_ in O_2_% using HSAC-supported Pt/C catalysts. A summary of the measured H_2_ in O_2_% at 0.5 A/cm^2^ is shown in (d). (e) The recorded H_2_ in O_2_% with time during the 12 h cell conditioning phase for cathodes (I/C ratio = 0.35) with HSAC-supported Pt/C catalysts. Ambient pressure at the anode is maintained for all measurements.

The H_2_ in O_2_% measurements for cathodes using HSAC support are similarly illustrated in Figure 4, covering the effects of Pt wt % in Pt/C (Figure 4a), Pt loading (Figure 4b), and ionomer content (Figure 4c). The previous findings from Figure 3 for Vulcan carbon-supported cathodes remain largely applicable to the HASC-supported cathodes with 40 and 50 wt % Pt catalysts. As depicted in Figure 4a, the cell with 50 wt % Pt exhibits a higher H_2_ in O_2_% than that with 40 wt % Pt at all of the three cathode pressures, confirming the conclusion that a higher Pt wt % leads to a higher H_2_ in O_2_%. The Pt loading effect, as seen in Figure 4b, shows a lower H_2_ in O_2_% for an increased Pt loading of 0.1 mg_Pt_/cm^2^ than that of 0.025 mg_Pt_/cm^2^ for cells with 50 wt % Pt catalyst, consistent with the results in Figure 3b. However, the difference in H_2_ in O_2_% between these two Pt loadings, especially at low current densities, is comparably smaller than that between Vulcan cathodes with a relatively high Pt wt % in Figure 3b, for example, the difference between two Pt loadings using 40 wt % Pt catalyst on Vulcan carbon. As for the cells with 40 wt % Pt catalyst on HSAC support in Figure 4b, the two curves of the different Pt loadings almost overlap each other, and their difference is even smaller. The reduced difference could be attributed to a larger ECSA provided by HSAC-supported Pt/C catalysts compared to those supported by Vulcan carbon. As a result, the benefit of a reduced hydrogen crossover rate through increasing the electrode roughness factor via an increased Pt loading becomes less evident in HSAC-supported catalysts. Figure 4c demonstrates the impact of ionomer content on H_2_ in O_2_%, showing an increase with increasing I/C ratio for the cathodes with 50 wt % Pt catalyst, in agreement with the findings in Figure 3c. In this case, a similarly 3 times increase in H_2_ in O_2_% at 0.5 A/cm^2^ is found, when the I/C ratio is increased from 0.35 to 0.69.

On the other hand, the measured results using 5 wt % Pt in Pt/C catalyst generally diverge from the previously observed trends. A notably higher H_2_ in O_2_% is recorded at an I/C ratio of 0.35 and a Pt loading of 0.025 mg_Pt_/cm^2^ in Figure 4a. It reaches a peak of approximately 2% at 2 A/cm^2^ under ambient cathode pressure. For its Vulcan carbon-supported cathode counterpart at the same conditions in Figure 3a, the corresponding value is only around 0.64%. Increasing the Pt loading to 0.1 mg_Pt_/cm^2^ does not yield a clear reduction in H_2_ in O_2_%, as shown in Figure 4b. Furthermore, an increase in ionomer content to an I/C ratio of 0.69 seems not to result in a substantial rise in H_2_ in O_2_%, which tends to remain within the range of about 1–1.5%. The cause for the anomaly with 5 wt % Pt catalyst is not yet fully understood. A possible explanation, as suggested by Figure 4e, might be degradation-related issues, indicated by an observed continuous increase of H_2_ in O_2_% during the cell 12-h conditioning phase at a constant current of 2 A/cm^2^. The H_2_ in O_2_% readings for most of the cells stabilize after around 1 h into the conditioning phase, with the exception of the cells using 5 wt % Pt catalyst. The corresponding data for cells with Vulcan-supported cathodes are shown in Figure S10. Literature indicates that for HSAC-supported Pt/C catalysts, the density of Pt particles located on the carbon surface increases with increasing Pt wt %.^22^ Consequently, the 5 wt % Pt catalyst demonstrates the most sparse Pt distribution on the surface, among those examined in this study. We speculate that the larger interparticle distance of Pt on the exterior of the carbon support for the 5 wt % Pt catalyst reduces the quenching rate of reactive oxygen species on the Pt surface, which leads to a more pronounced rate of degradation.^26^ Alternatively, it could also be due to mass transfer limitations arising from the complex combined effects of a large Pt interparticle distance, a low amount of surface Pt, and the bottleneck structure in the pores of the highly porous HSAC support.

Figure 4d presents a summary of H_2_ in O_2_% at 0.5 A/cm^2^ for cells using HSAC-supported cathodes, revealing trends that are partially similar yet distinct from those with Vulcan cathodes in Figure 3d. At an I/C ratio of 0.35, reducing the Pt wt % from 50 to 40 wt % decreases the H_2_ in O_2_%, whereas a further reduction to 5 wt % induces an increase, potentially because of degradation or mass transfer limitations (as explained above). For both 40 and 50 wt % Pt catalysts, a decrease in the I/C ratio from 0.69 to 0.35 leads to a reduced H_2_ in O_2_%, while varying the Pt loading between 0.025 and 0.1 mg_Pt_/cm^2^ yields a comparatively minor effect, possibly due to the high porosity of HSAC. Overall, to attain a minimal H_2_ in O_2_% at a reduced Pt loading of 0.025 mg_Pt_/cm^2^, a cathode configuration consisting of 40 wt % Pt in Pt/C and an I/C ratio of 0.35 is concluded to be optimal.

**Figure 5:**
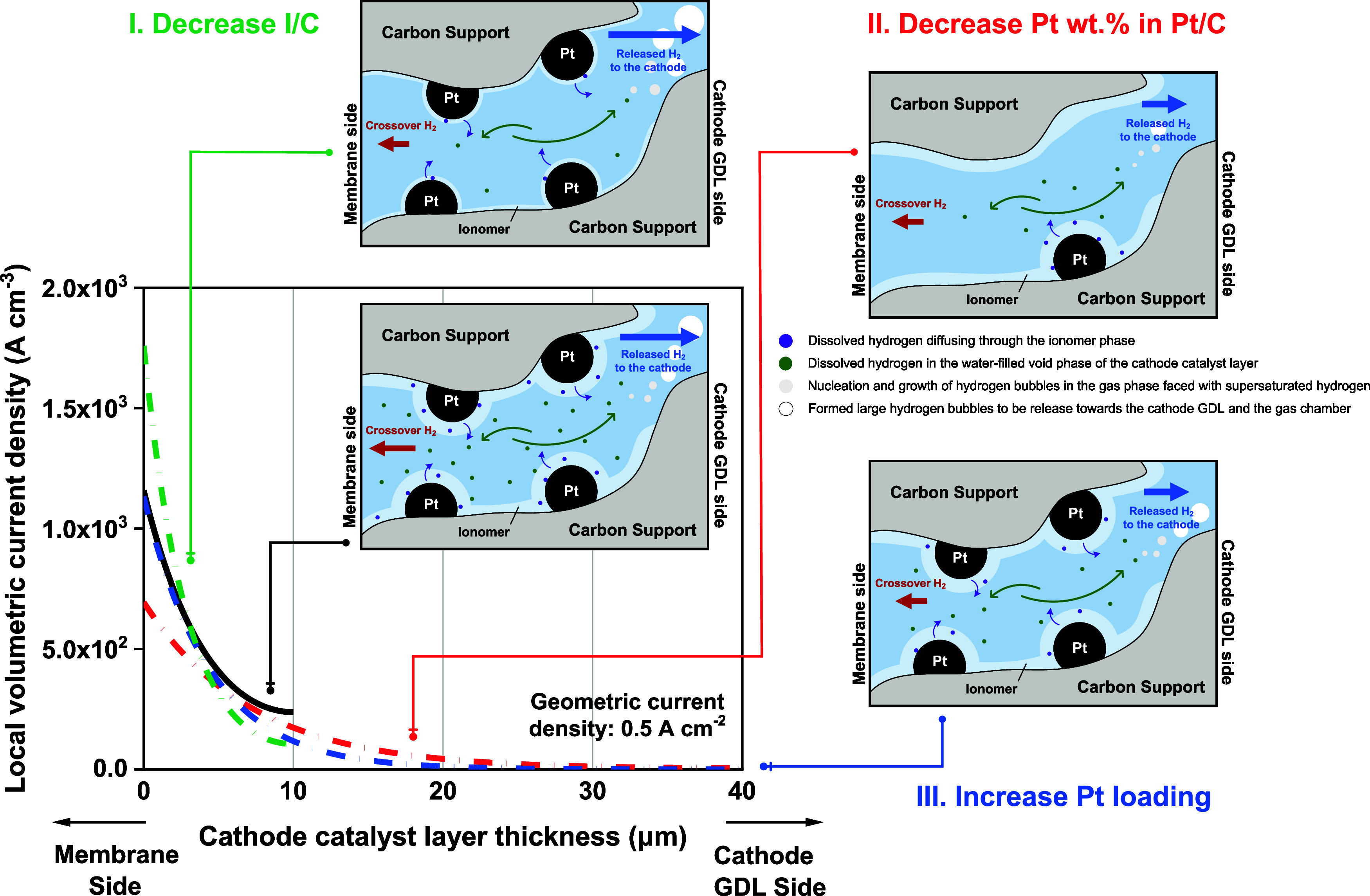
Local volumetric current density within the cathode catalyst layer at a geometric current density of 0.5 A/cm^2^ as determined by a set of modeled experimental conditions and its associated variations. The black line represents the base scenario of a 10 μm thick cathode catalyst layer, with 40 wt % Pt in Pt/C catalyst, a Pt loading of 0.025 mg_Pt_/cm^2^, and an I/C ratio of 0.69. The schematics show the local hydrogen concentration profiles close to the membrane–electrode interface.

Drawing on the experimental findings from Vulcan and HSAC cathodes, we aim to elucidate the impact of structural parameters of the cathode catalyst layer on the rate of hydrogen crossover in an operating cell. This is achieved by examining the local hydrogen concentration profile within the catalyst layer near the membrane–electrode interface. As shown in Figure 5, the local volumetric current density, expressed in A/cm^3^, is plotted as a function of its position along the thickness of the cathode catalyst layer. The profile is derived based on a kinetic model using parameters that closely mirror the experimental conditions (cf. Supporting Information).^10,19,27^ A geometric current density of 0.5 A/cm^2^ is chosen. The base profile, shown by the black line, represents a 10 μm thick cathode catalyst layer, with 40 wt % Pt in Pt/C catalyst, a Pt loading of 0.025 mg_Pt_/cm^2^, and an I/C ratio of 0.69. From this baseline, we analyze three scenarios involving their respective change in the cathode structural parameters: I. decreasing the I/C ratio from 0.69 to 0.35; II. decreasing Pt wt % in Pt/C from 40 wt % to 10 wt %; III. increasing the Pt loading from 0.025 to 0.1 mg_Pt_/cm^2^. It is assumed that for scenarios II and III, these changes both lead to a 4-fold increase in the thickness of the cathode catalyst layer.

Consequently, the following effects are discussed: In scenario I, as shown by the green line, reducing the I/C ratio leads to an increase in the local volumetric current density at the membrane–electrode interface. This current density then experiences a more rapid decline than the base profile and eventually ends at a lower level at positions far from the membrane–electrode interface. The thickness of the ionomer layer on the catalyst particles is considered to be smaller than that in the base profile, which shortens the diffusion path of produced hydrogen required to reach the surrounding open space. In addition, there is an increase in the hydraulic permeability and volume-specific pore surface area under these conditions.^15^ Therefore, despite a volumetric increase in hydrogen production at the membrane–electrode interface, the combined effect results in an enhanced mass transfer of the dissolved produced hydrogen and thereby a reduced local hydrogen concentration under the specified experimental settings. This accounts for the observed decrease in H_2_ in O_2_% with a lower I/C ratio. On the other hand, it is important to note if the I/C ratio keeps decreasing even further, at some point, the measured H_2_ in O_2_% is likely to reverse its decreasing trend and start to increase. Because with a further reduced I/C ratio, the local volumetric current density is expected to continue increasing at the membrane–electrode interface. This will eventually become significant compared to the other factors, resulting in an effectively increased supersaturated hydrogen concentration at the interface and therefore an increased hydrogen crossover rate.

In scenario II, indicated by the red line, a decrease in Pt wt % causes a considerable decrease in the local volumetric current density at and near the membrane–electrode interface, extending the reaction zone due to the 4-fold increase in the catalyst layer thickness. For example, compared to the base profile, the current density at the interface (*x* = 0) drops from 1158 to 694 A/cm^3^, making an approximate 1.7 times decrease in volumetric hydrogen production. This reduction is assumed to serve as a primary factor in effectively diluting the local hydrogen concentration and therefore resulting in a decreased H_2_ in O_2_%.

In scenario III, as depicted with the blue line, increasing the Pt loading from 0.025 to 0.1 mg_Pt_/cm^2^ increases the electrode roughness factor by 4 times, consequently expanding the reaction zone. Compared to the base profile, the current density at the membrane–electrode interface (*x* = 0) is almost unchanged, yet it decreases faster farther away from the membrane, indicating a more rapid decrease in volumetric hydrogen production relative to the position within the catalyst layer. This dilution effect, predominately occurring near but not directly at the interface, leads to a reduced local hydrogen concentration and, consistent with experimental observation, particularly in electrodes with Vulcan carbon support, a decrease in H_2_ in O_2_%.

### Hydrogen Mass Transfer Coefficient in Cathode Components

To gain a quantitative understanding of the mass transfer processes of the produced hydrogen within the cell’s cathode components, the hydrogen mass transfer coefficient is calculated based on the measured H_2_ in O_2_%. The H_2_ in O_2_% (*x*_H_2_ in O_2__), under the assumption of negligible oxygen permeation from the anode to the cathode as it is at least twice lower than the hydrogen permeation,^28^ can be linked to the crossover hydrogen flux (*N*_H_2__^Perm^):
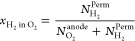
3Here, *N*_O_2__^anode^, representing the oxygen flux generated at the anode, is calculated via Faraday’s laws:
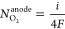
4where *i* is the current density and *F* is the Faraday constant. Combining eqs 3 and 4, the crossover hydrogen flux in an operating cell is experimentally determined as
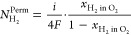
5On the other hand, applying hydrogen mass conservation at the cathode, the produced hydrogen flux (*N*_H_2__^cathode^) is equal to the sum of the hydrogen permeating through the membrane (*N*_H_2__^Perm^) and the hydrogen transported to the cathode compartment (*N*_H_2__^trans^):
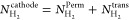
6

Applying Faraday’s laws for hydrogen generation (eq 7) and considering a diffusion-driven process (according to Fick’s first law of diffusion) for the crossover hydrogen flux (eq 8) and a conventional mass transport model characterized by a global mass transfer coefficient (*k*) for the hydrogen moving toward the cathode compartment (eq 9), Trinke et al. developed an expression for the local hydrogen concentration (*c*_H_2__^*^) at the cathode near the catalyst site (eq 10):^14^
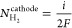
7
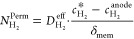
8
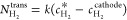
9
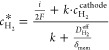
10Here, *D*_H_2__^eff^ is the effective diffusion coefficient of hydrogen in the membrane, δ_mem_ is the thickness of the membrane, and *c*_H_2__^cathode^ is the equilibrium concentration of the dissolved hydrogen at the cathode.

Substituting eq 10 into eq 8, assuming negligible equilibrium dissolved hydrogen concentration at the anode (*c*_H_2__^anode^ ≈ 0), yields an expression of the crossover hydrogen flux:^14^
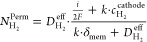
11This expression for the crossover hydrogen flux can be approximated as a linear function of the current density *i*, assuming all of the other variables remain constant or are not significantly affected by the current density change within the range investigated:
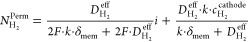
12The slope, as specified in eq 12, can be obtained through linear regression of the experimentally determined crossover hydrogen flux via eq 5, for calculating the global mass transfer coefficient of hydrogen, as
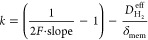
13

**Figure 6:**
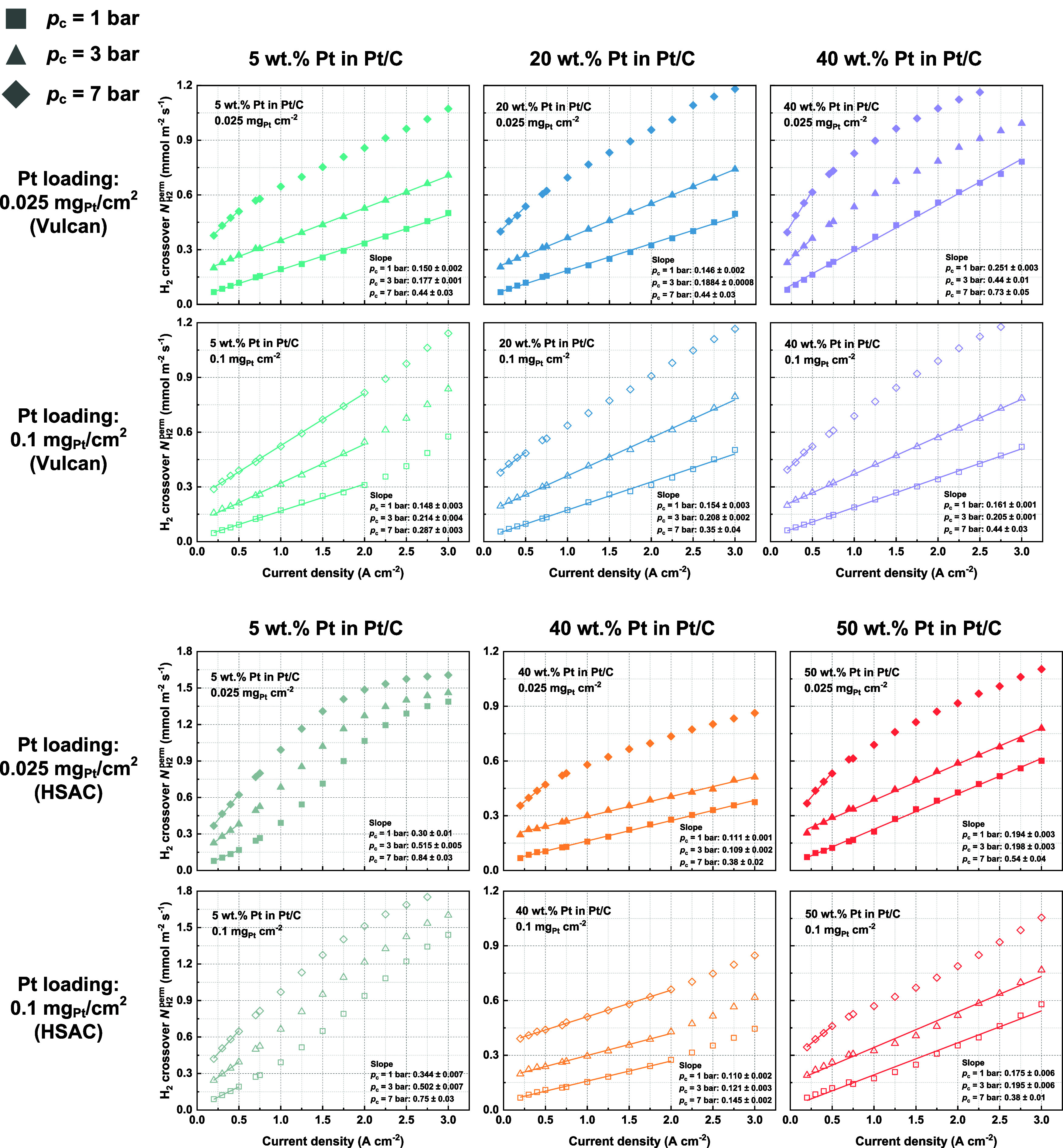
Hydrogen crossover flux as a function of current density for Vulcan carbon and HSAC-supported Pt/C catalysts (I/C ratio = 0.35) and the corresponding linear regression results. For all measurements, an ambient anode pressure is maintained.

The global mass transfer coefficient describes the ease of transport of hydrogen from the catalyst surface to the cathode flow field. Considering a consistent cell setup across all experiments, the observed changes in the calculated mass transfer coefficient are therefore largely attributed to variations in the cathode catalyst layer structure. Figure 6 presents the crossover hydrogen flux results at an I/C ratio of 0.35, measured at three cathode pressure levels. A linear fit of the data is performed within the estimated linear range between 0.2 and 0.5, 2.0, or 3.0 A/cm^2^, with an *R*^2^ value greater than 0.985. Deviations from the linear trend in the data points at higher current densities may be due to various factors, for instance, the center of the reaction front moving closer to the membrane or an increased hydrogen back-transport because of a higher electro-osmotic drag of water at high current density.^25^Figure S11 includes the measurement results at an I/C ratio of 0.69, with selected linear regression conducted at ambient cathode pressure.

**Figure 7:**
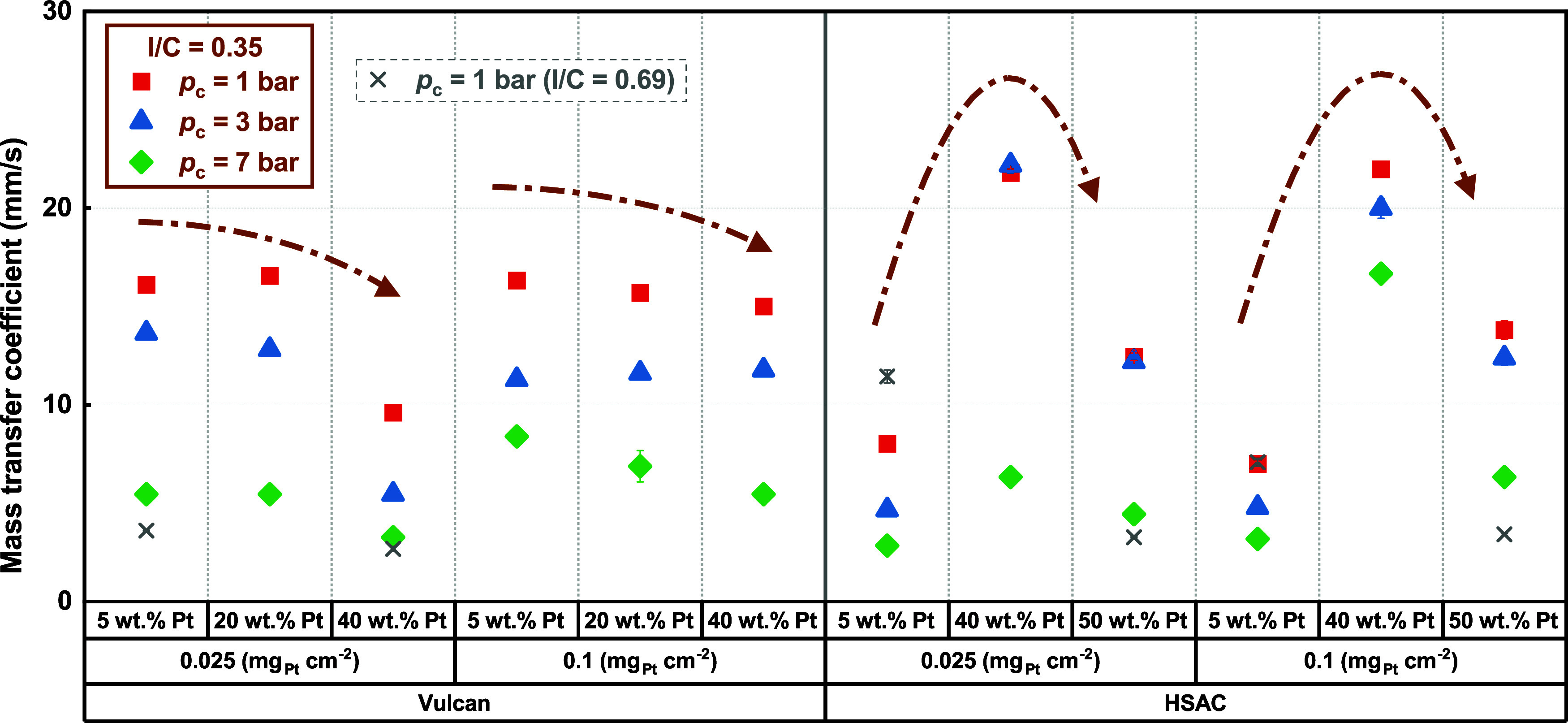
Calculated hydrogen mass transfer coefficients in the cell’s cathode components for various cathode catalyst layer configurations. Ambient pressure at the anode is maintained.

The calculated hydrogen mass transfer coefficients for the investigated cathode configurations are summarized in Figure 7. A larger value in the mass transfer coefficient indicates a more efficient release of the produced hydrogen to the cathode flow field, which leads to a reduced local hydrogen concentration and a lower crossover hydrogen flux. Generally, it is observed that with an increase in Pt wt % or I/C ratio, the mass transfer coefficient declines, except in the case of 5 wt % Pt with HSAC support, aligning with our aforementioned findings. Between the two different Pt loading levels, the coefficients are mostly similar, while the cathode of 40 wt % Pt with Vulcan support shows a relatively larger increase as the Pt loading increases from 0.025 to 0.1 mg_Pt_/cm^2^. When comparing Vulcan and HSAC cathodes at a reduced Pt loading of 0.025 mg_Pt_/cm^2^, the HSAC cathode with 40 wt % Pt and an I/C ratio of 0.35 outperforms the others, demonstrating the highest mass transfer coefficient of over 20 mm/s at ambient cathode pressure. This is likely a result of the synergistic effects of HSAC’s higher available surface area with a finer Pt particle distribution compared to Vulcan carbon, a relatively lower Pt wt % than the 50 wt % Pt HSAC, and a low ionomer content.

The hydrogen mass transfer coefficient obtained in this study generally aligns with the order of magnitude reported in the literature under similar conditions.^15^ Furthermore, an increase in cathode pressure is observed to correspond to a decrease in the mass transfer coefficient, a phenomenon also reported by Omrani et al.^29^

## Conclusions

This study reports the effects of reducing cathode Pt loading to an ultralow level of 0.025 mg_Pt_/cm^2^ in a PEM water electrolysis cell and concurrently aims to minimize the cell hydrogen crossover by optimizing the design parameters of the cathode catalyst layer. Key variables including Pt wt % in Pt/C, the type of carbon support, and the ionomer content were systematically examined. The analysis of the measured H_2_ in O_2_% data indicates that a lower Pt wt % and I/C ratio generally contribute to a reduced hydrogen permeation rate. In contrast, a decrease in Pt loading may lead to an opposite effect, especially in cathodes using Vulcan carbon-supported catalysts. These observations were elucidated by looking into the local volumetric current density and the changes in local hydrogen concentration near the membrane–electrode interface. The effective hydrogen mass transfer coefficient in the cathode components was calculated, and the results were found to be largely consistent with experimental findings. Notably, at a reduced Pt loading of 0.025 mg_Pt_/cm^2^, an electrolysis cell featuring the cathode configuration of 40 wt % Pt on HSAC support with an I/C ratio of 0.35 is demonstrated to yield the highest hydrogen mass transfer coefficient. Consequently, this specific configuration is considered as optimal for a low hydrogen crossover cathode at an ultralow Pt loading level.

## Experimental Section

### Pt/C Catalyst

Six different commercial Pt/C catalysts were used in this study. For Pt/C with Vulcan XC-72 (denoted as Vulcan) carbon support, we selected catalyst powders with nominal Pt contents of 5, 20, and 40 wt % in Pt/C (Fuel Cell Store). For Pt/C with high surface area carbon (denoted as HSAC), catalyst powders with nominal Pt contents of 5 wt % (actual 5.1 wt %; TEC10E05E), 40 wt % (actual 37.4 wt %; TEC10E40E), and 50 wt % (actual 46.1 wt %; TEC10E50E) (Tanaka) were chosen.

### Preparation of the Catalyst-Coated Membrane (CCM)

Catalyst-coated membranes (CCM) with an active area of 25 cm^2^ were made by spray coating the prepared catalyst ink on to a commercial Nafion 212 membrane (Nafion NR212, Ion Power) using an automatic spray coating machine (ExactaCoat, Sono-tek) with an ultrasonic nozzle. Catalyst ink was filled into a syringe equipped with a magnetic stirring bar, and the flow rate was fixed at 0.05 mL/min. The Nafion membrane was clamped between two polyoxymethylene half-frames and installed on a large metal frame, which was placed on a hot plate at 50 °C in a spray coating machine. In order to achieve the target catalyst metal loading, a small reference sheet of poly(tetrafluoroethylene) (PTFE) was taped onto the metal frame and periodically weighed.

The catalyst ink was prepared by mixing specific cathode or anode catalyst powder, milli-Q water, isopropanol, and Nafion dispersion (Nafion D521CS, 1100 EW at 5 wt % weight percent, Ion Power). The mixture was subjected to at least 15 min of sonication before adding the Nafion dispersion and an additional 15 min sonication after adding the Nafion dispersion to form the final ink. For the anode catalyst ink, IrO_2_ supported on TiO_2_ (74.1 wt % Ir, Elyst Ir75 0480, Umicore) was used as the catalyst powder. It was mixed with water, isopropanol, and Nafion dispersion in a weight ratio of 2.3:0.6:1. The Ir loading was adjusted to 2.0 mg_Ir_/cm^2^. For the cathode, various Pt/C catalyst powders were used with two Pt loadings of 0.1 and 0.025 mg_Pt_/cm^2^. In the case of an ionomer–to-carbon (I/C) ratio of 0.35, water, isopropanol, and Nafion dispersion were mixed based on a weight ratio of 2.4/3.1/1. For a higher I/C ratio of 0.69, the respective weight ratio is 1.2/1.6/1.

### Electrolysis Cell Measurement

The prepared CCM was assembled in an electrolysis cell, which was then mounted onto a home-built electrolysis testbench for electrochemical measurements at 80 °C, as described previously.^30^ The anode water was circulated at a flow rate of ∼300 mL/min and purified in an ion-exchange bed during each pass. The anode pressure was kept at ambient throughout the study, while three levels of cathode pressures (1, 3, and 7 bar) were employed. For each of the new CCMs, the cell was conditioned at 2 A/cm^2^ for 12 h before carrying out polarization and hydrogen crossover measurements. To evaluate the rate of cell hydrogen crossover, constant currents between 0.2 and 3 A/cm^2^ were held for a total of 15 data points. Because of the lower rate of oxygen production at lower current densities, the time required to stabilize the measured H_2_ in O_2_% differed. A longer holding time was used for lower current densities.

### Physical Characterization

Scanning electron microscopy (SEM, Zeiss NVision 40) was performed on CCMs for the cathode catalyst layer surface morphology and cross-sectional thickness measurements. The cross-section SEM samples were prepared by cryofracturing with liquid nitrogen. The thickness of the catalyst layer was determined based on an average of measurements from at least 5 different locations in cross-sectional images using ImageJ.

Selected Pt/C catalyst powders were chosen for transmission electron microscopy (TEM, JEOL JEM-ARM200F, 200 kV) images. The powder was dispersed in Milli-Q water under sonication, with drops of the mixture then transferred onto a Cu-grid and dried.

## Supporting Information Available

SEM images, electrochemical performance results, measured H_2_ in O_2_% data with selected repeats and the calculated hydrogen crossover rates for the designated samples, and details of local volumetric current density calculation in the cathode catalyst layer. (PDF)
