# Supplementary material for: Cathode Catalyst Layer Design in PEM Water Electrolysis toward Reduced Pt Loading and Hydrogen Crossover
Source: ACS Appl Mater Interfaces. 2024 Apr 23;16(18):23265–77. doi: 10.1021/acsami.4c01827 (PMC11082850; doi:10.1021/acsami.4c01827)
Supplement: Supplementary file 1 — am4c01827_si_001.pdf [file am4c01827_si_001.pdf]

Supporting Information: Cathode Catalyst Layer Design in PEM Water Electrolysis toward Reduced Pt Loading and Hydrogen Crossover

Zheyu Zhang[1], Axelle Baudy[1,†], Andrea Testino[2,3], and Lorenz Gubler[1,]*

[1] Electrochemistry Laboratory, Paul Scherrer Institut, 5232 Villigen PSI, Switzerland

[2] Laboratory for Sustainable Energy Carriers and Processes, Paul Scherrer Institut, 5232 Villigen PSI, Switzerland

[3] STI SMX-GE, École Polytechnique Fédérale de Lausanne, 1015 Lausanne, Switzerland

* Corresponding Author: lorenz.gubler@psi.ch

† Present address: Univ. Grenoble Alpes, Univ. Savoie Mont Blanc, CNRS, Grenoble INP (Institute of Engineering and Management), LEPMI, 38000 Grenoble, France

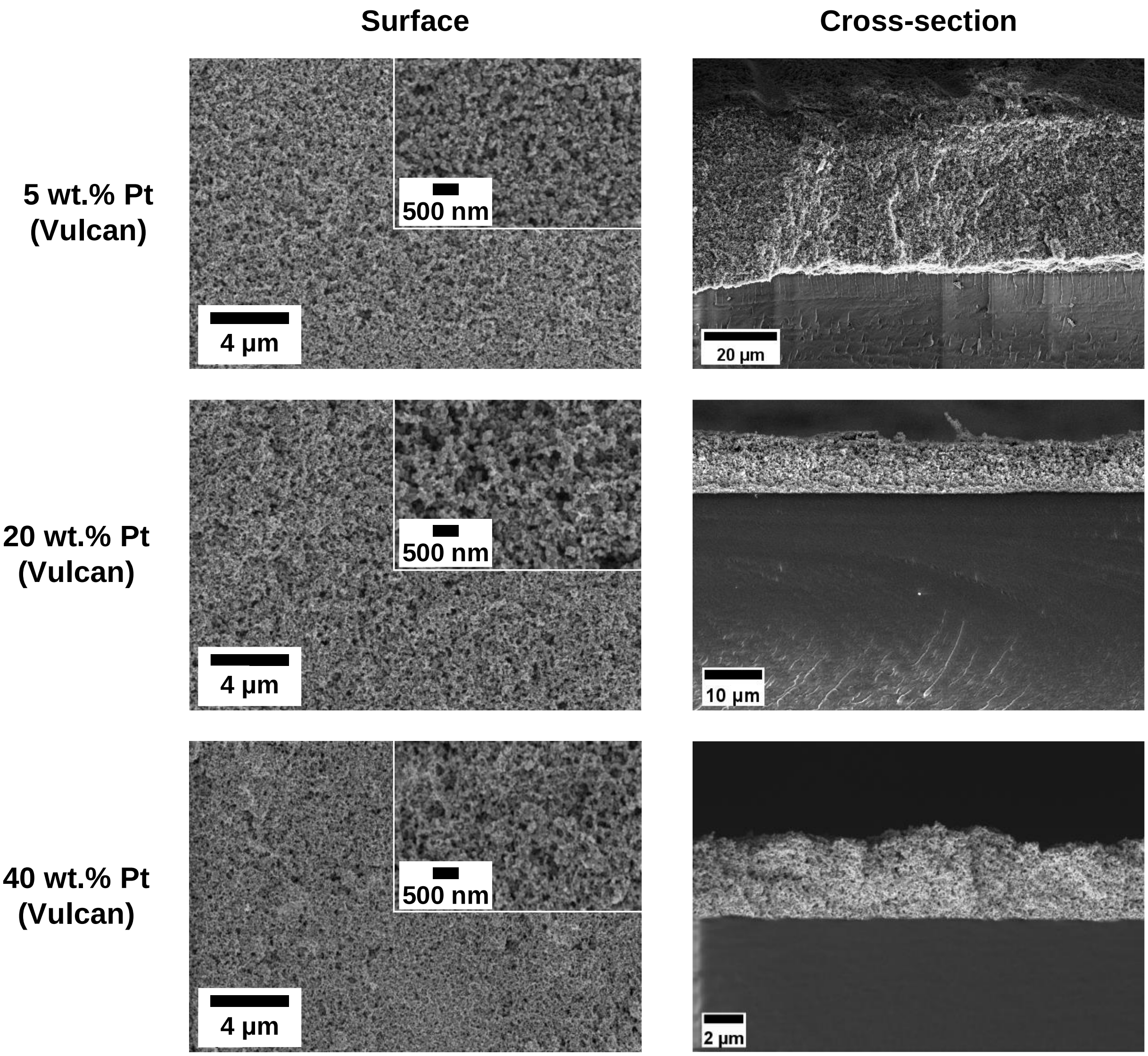

Figure S1. SEM surface and cross-sectional images for cathode catalyst layers (0.1 mg_{Pt}/cm^2 and an I/C ratio of 0.35) with Vulcan carbon-supported Pt/C catalyst.

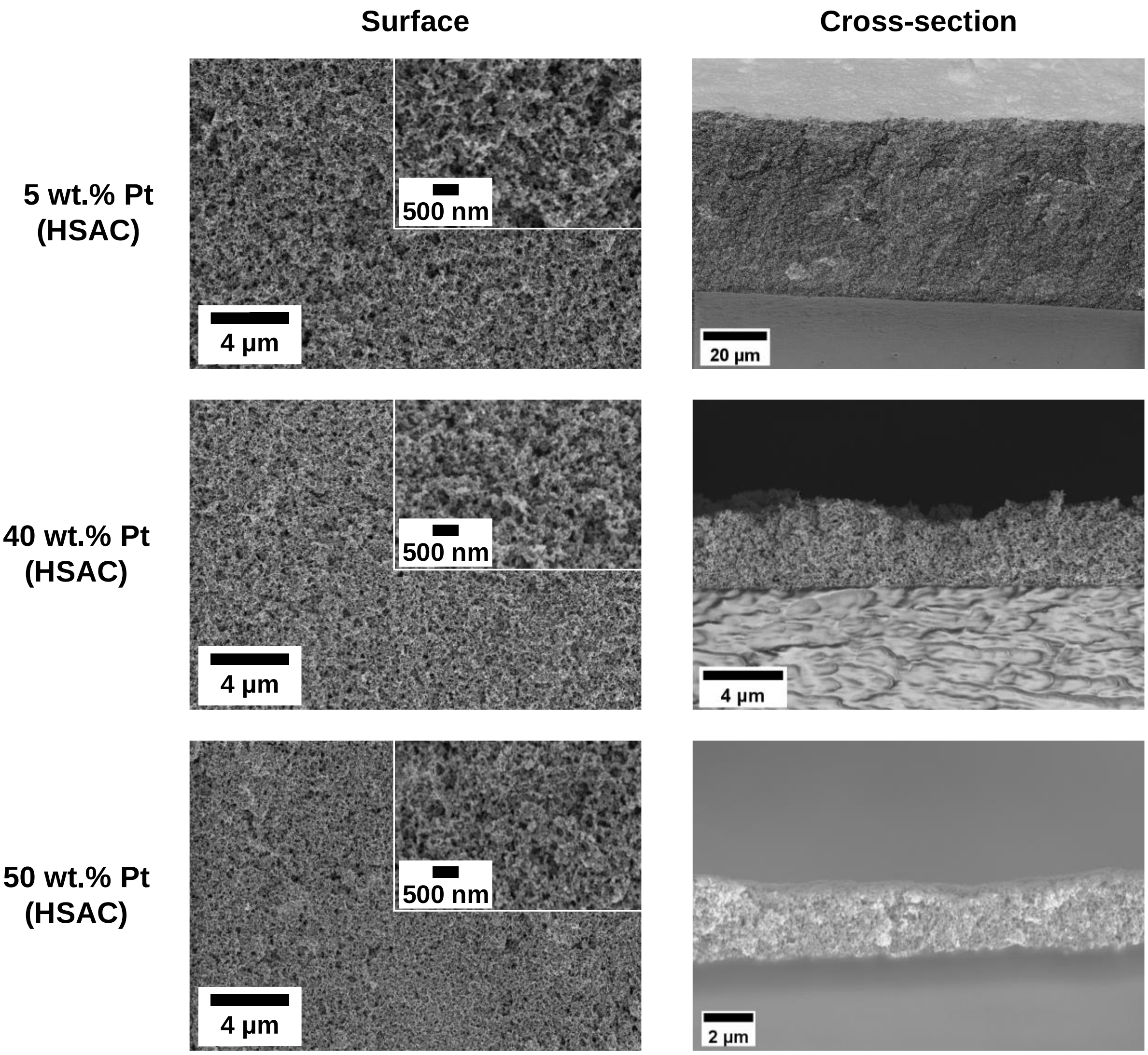

Figure S2. SEM surface and cross-sectional images for cathode catalyst layers (0.1 mg_{Pt}/cm^2 and an I/C ratio of 0.35) with HSAC-supported Pt/C catalyst.

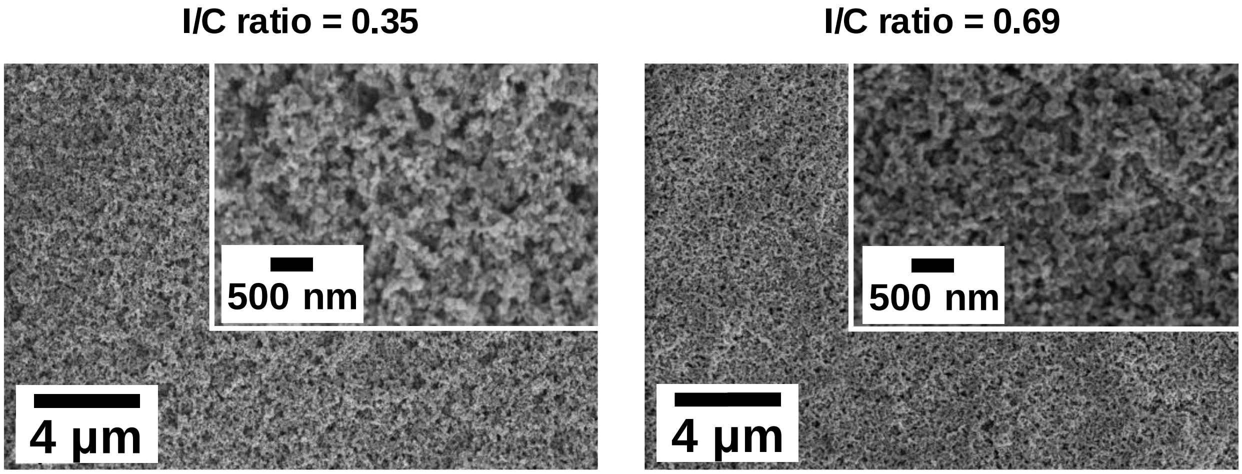

Figure S3. SEM images showing comparison of surface morphology for cathode catalyst layers (5 wt.% Pt on Vulcan carbon support and 0.025 mg_{Pt}/cm^2) with an I/C ratio of 0.35 and 0.69.

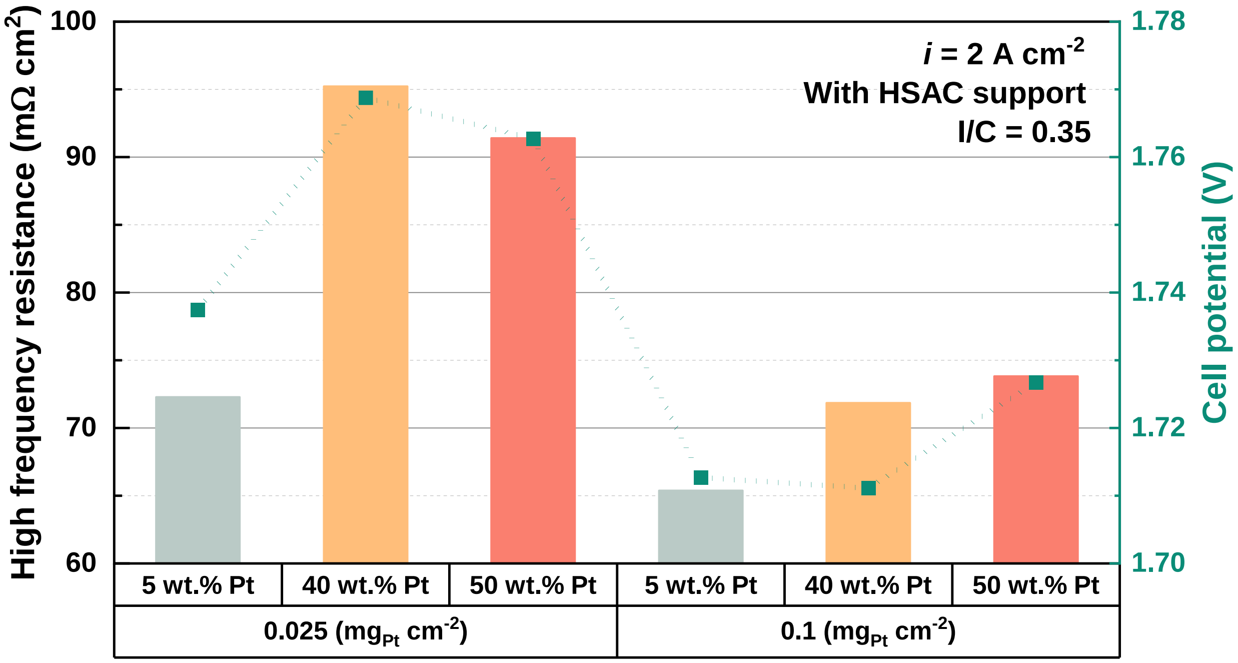

Figure S4. A summary of the high frequency resistance and cell potential at 2 A/cm^2 (I/C ratio = 0.35) for HSAC-supported Pt/C catalysts. Both the cathode and anode pressures are maintained at ambient conditions. The observed deviation from the general trend in cathodes with 40 and 50 wt.% Pt in Pt/C at 0.025 mg$_{Pt}$/cm^2 may be attributed to the similarity in thickness of these catalyst layers, both in the range of ~ 1 μm. The small difference in the thickness could result in an effect that is less pronounced than the CCM sample-to-sample variations.

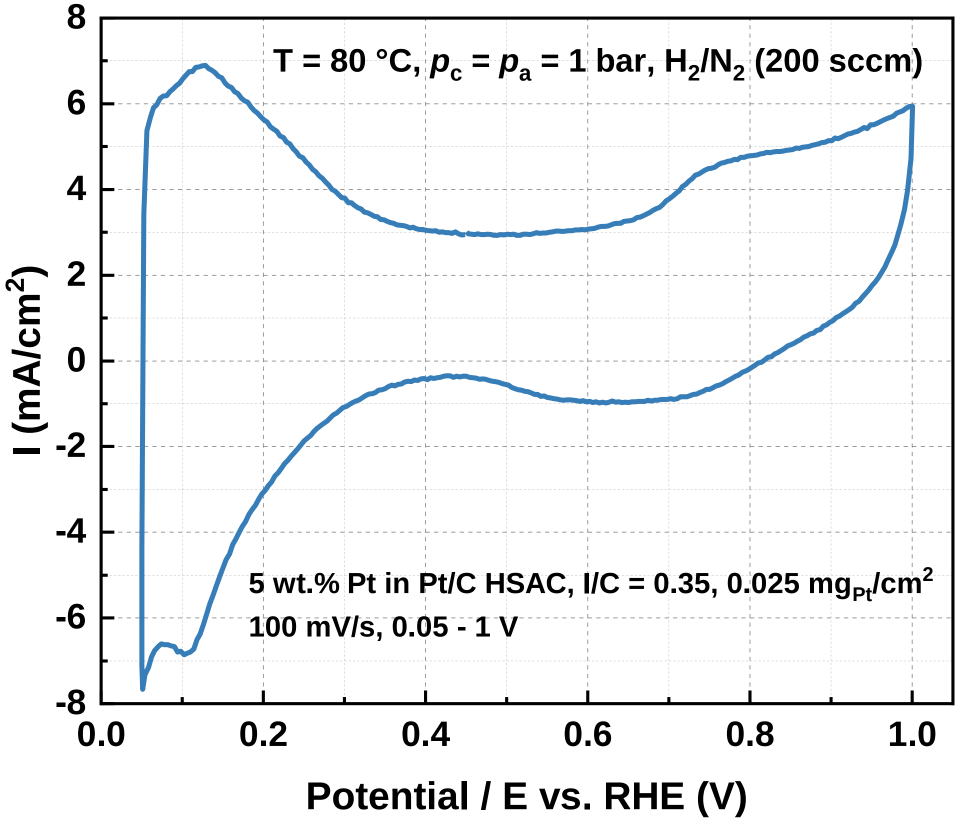

Figure S5. Cyclic voltammograms recorded on a symmetrical CCM with both sides using 5 wt.% Pt in Pt/C catalyst on HSAC support. The measurement was conducted in an electrolysis cell setup, where the reference side was purged with fully humidified H_2 and the working electrode side was immersed in circulating liquid water with saturated N_2.

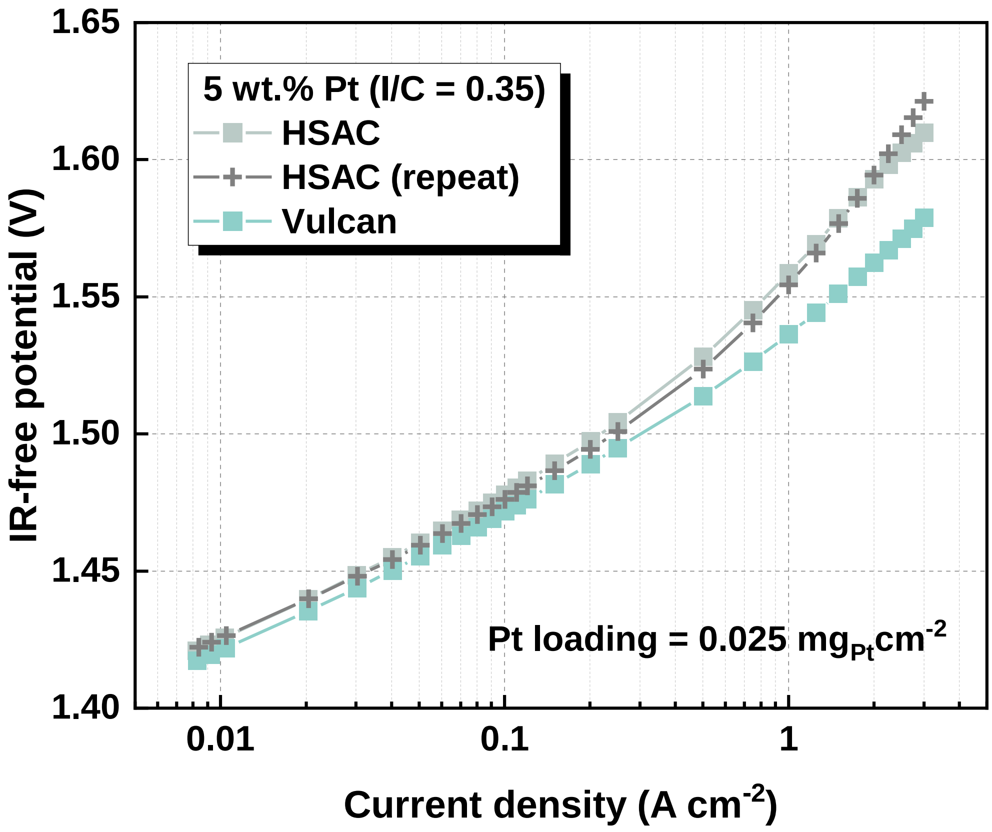

Figure S6. IR-free potentials for 5 wt.% Pt in Pt/C catalysts on HSAC and Vulcan carbon support at a Pt loading of 0.025 mg_{Pt}/cm^2 and an I/C ratio of 0.35. Both the cathode and anode pressures are maintained at ambient conditions.

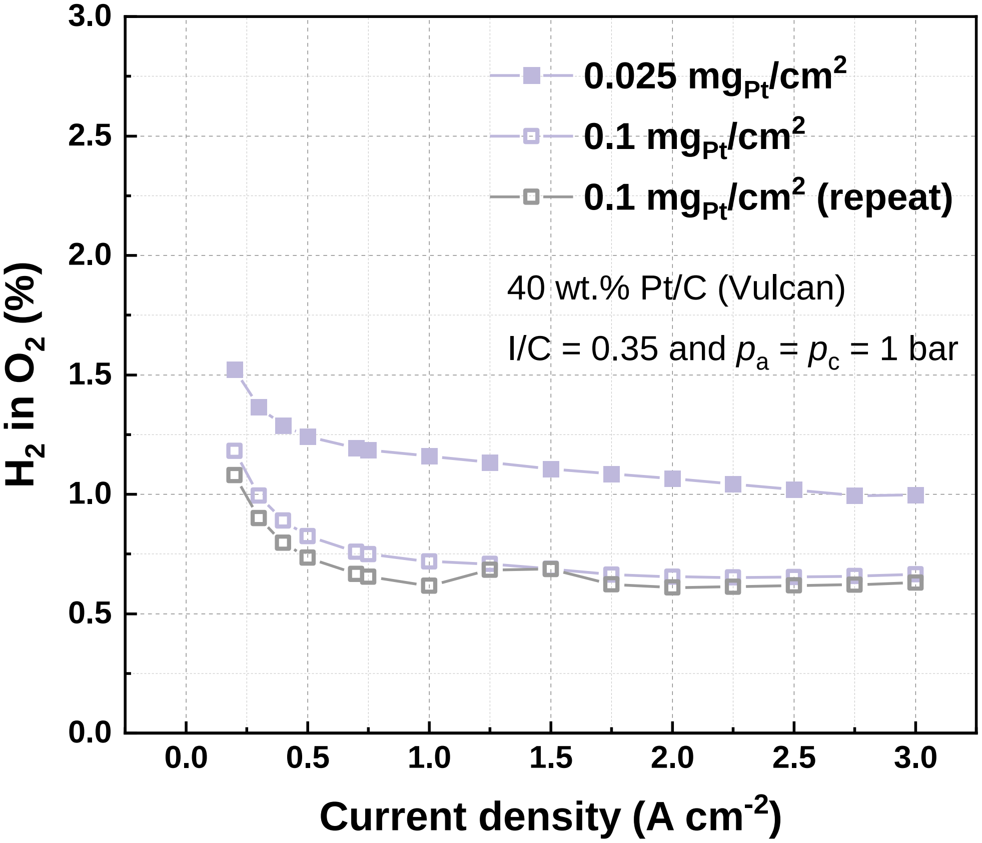

Figure S7. Effect of Pt loading on the measured H_2 in O_2% using Vulcan-carbon supported 40 wt.% Pt/C catalyst, with a repeated measurement, at ambient pressure conditions. At 0.5 A/cm^2, the sample standard deviation for the measurements repeated is calculated to be 8% relative to the mean.

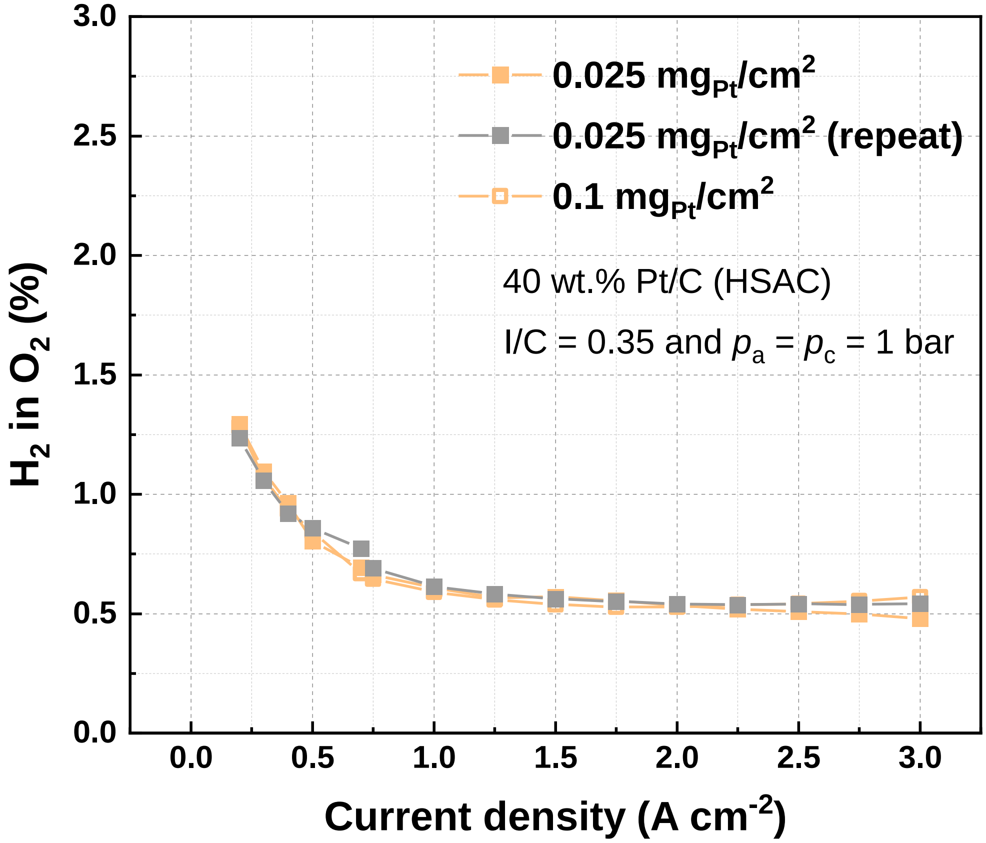

Figure S8. Effect of Pt loading on the measured H_2 in O_2% using HSAC supported 40 wt.% Pt/C catalyst, with a repeated measurement, at ambient pressure conditions. At 0.5 A/cm^2, the sample standard deviation for the measurements repeated is calculated to be 5% relative to the mean.

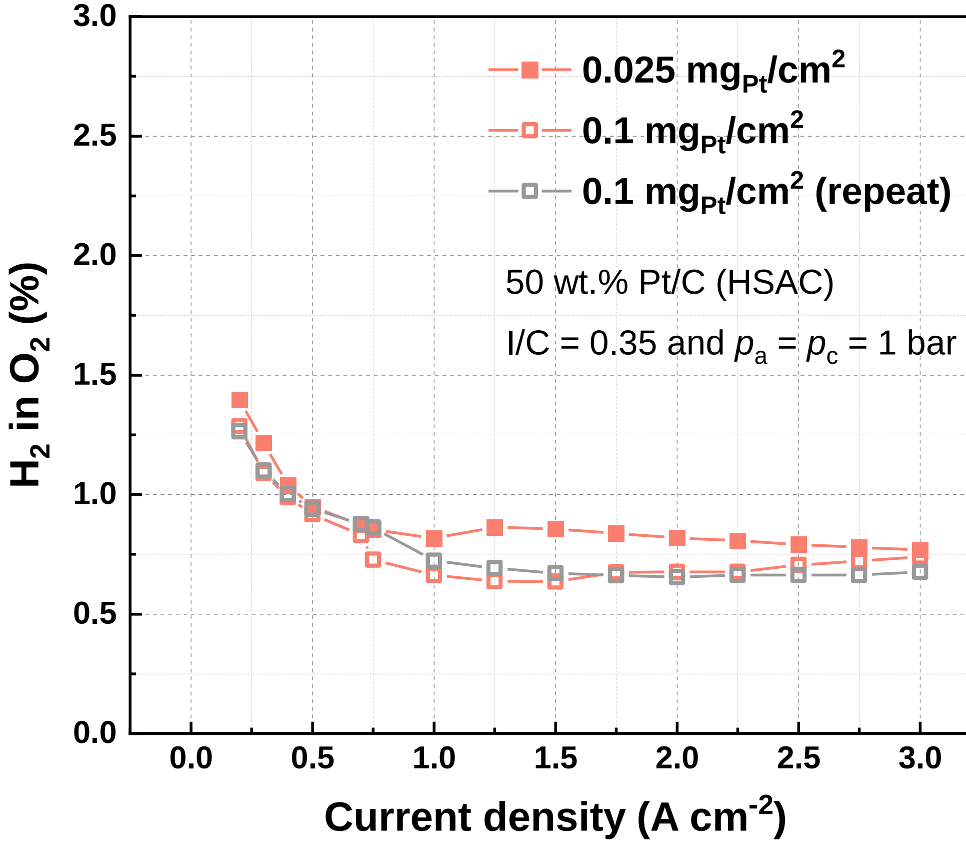

Figure S9. Effect of Pt loading on the measured H_2 in O_2% using HSAC supported 50 wt.% Pt/C catalyst, with a repeated measurement, at ambient pressure conditions. At 0.5 A/cm^2, the sample standard deviation for the measurements repeated is calculated to be 2% relative to the mean.

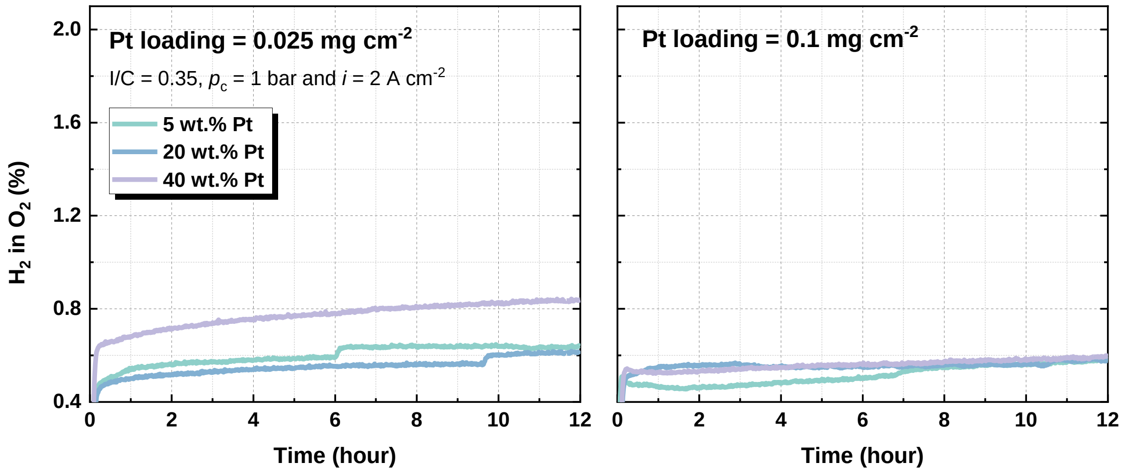

Figure S10. The recorded H_2 in O_2% with time during the 12-hour cell conditioning phase for cathodes with Vulcan carbon-supported Pt/C catalysts (I/C ratio = 0.35).

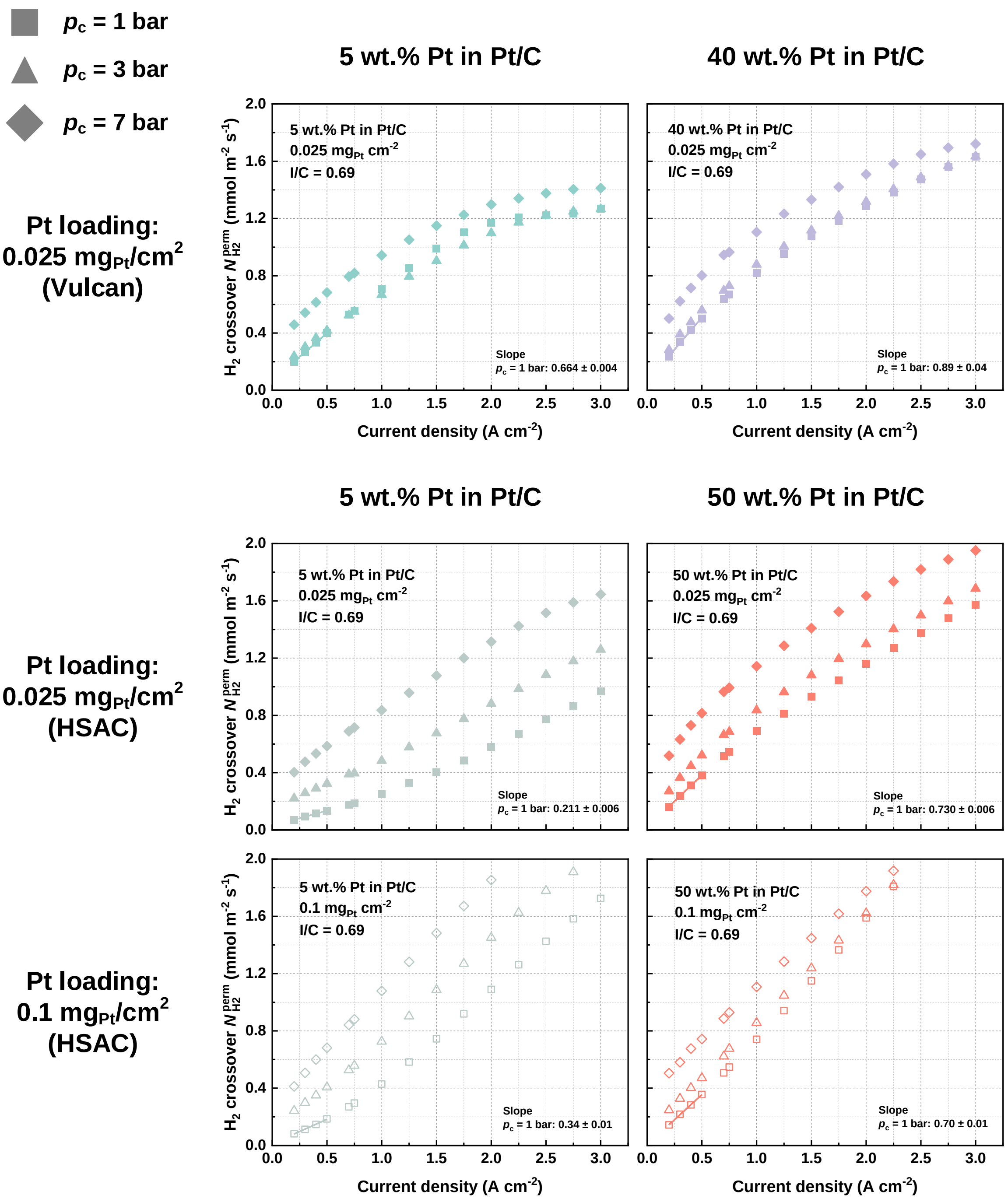

Figure S11. Hydrogen crossover flux vs. current density for Vulcan carbon and HSAC-supported Pt/C catalysts (I/C ratio = 0.69), and the corresponding selected linear regression results at ambient cathode pressure. For all measurements, an ambient anode pressure is maintained.

Calculation of the local volumetric current density in the cathode catalyst layer

The local volumetric current density (i_v, in A/cm^3) in the catalyst layer is determined assuming linear kinetics of the HER:

$$i_v = \frac{\eta_{\mathrm{HER}}}{\delta \cdot R_{\mathrm{K,HER}}} \quad (1)$$

where δ is the cathode catalyst layer thickness, η_{HER} is the kinetic overpotential and $R_{\mathrm{K,\,HER}}$ is the charge transfer resistance for the HER. As described by Bernt et al., $R_{\mathrm{K,\,HER}}$ can be expressed as[1]:

$$R_{\mathrm{K,HER}} = \frac{R \cdot T}{(\alpha_{\mathrm{a}} + \alpha_{\mathrm{c}}) \cdot F \cdot L_{\mathrm{Pt}} \cdot A_{\mathrm{Pt}} \cdot i_{0,\mathrm{HER}}} \quad (2)$$

where R is the gas constant, T is the temperature, (α_{a} + α_{c}) is the sum of the anodic and cathodic transfer coefficients, F is the Faraday constant, L_{Pt} is the Pt loading, A_{Pt} is the specific Pt surface area, and $i_{0,\,\mathrm{HER}}$ is the exchange current density of the HER. The estimated values of these parameters are listed in Table 1. The referenced values of A_{Pt} are quoted from the manufacturer (Fuel Cell Store) for 20 wt.% and 5 wt.% Pt in Pt/C on Vulcan carbon XC-72.

The kinetic overpotential η_{HER} is calculated by solving a second-order ordinary differential equation with two defined boundary conditions, similar to the derivations made by Thompson et al. for the hydrogen oxidation reaction[2]:

$$\eta_{HER}|_x = \frac{i}{s \cdot \kappa_{\mathrm{eff}}} \cdot \frac{e^{s(\delta - x)} + e^{-s(\delta - x)}}{e^{s \cdot \delta} - e^{-s \cdot \delta}} = \frac{i}{s \cdot \kappa_{\mathrm{eff}}} \cdot \frac{\cosh\left(s(\delta - x)\right)}{\sinh\left(s \cdot \delta\right)} \quad (3)$$

Here, x is the location variable along the thickness of the cathode catalyst layer (where the origin is set at the interface between the membrane and the cathode catalyst layer, and the positive direction extends towards the cathode GDL), i is the geometric current density, κ_{eff} is the effective proton conductivity of the cathode catalyst layer, and s is a kinetic parameter:

$$s = \left[\frac{1}{\kappa_{\mathrm{eff}} \cdot \delta} \cdot \frac{1}{R_{\mathrm{K,HER}}}\right]^{\frac{1}{2}} \quad (4)$$

The effective proton conductivity κ_{eff} can be calculated by:

$$\kappa_{\mathrm{eff}} = \frac{1}{\rho_{\mathrm{H+,cath}}} \quad (5)$$

where $\rho_{\mathrm{H+,\,cath}}$ is the effective proton resistivity of the cathode catalyst layer, and is estimated to be ~ 25 Ohm·cm for an I/C ratio of 0.69 and ~ 60 Ohm·cm for an I/C ratio of 0.35.[3] A homogenous catalyst layer composition is assumed and the local Pt/ionomer interfaces effect is not considered.

Table 1. Parameters used for calculation of the local volumetric current density profiles in the cathode catalyst layer.

Parameter \ Profile	Base	Scenario I	Scenario II	Scenario III
i (A cm^{-2})	0.5			
T (K)	353			
$i_{0,\,\mathrm{HER}}$ (mA cm^{-2}) [1]	250			
$\alpha_{\mathrm{a,\,HER}} + \alpha_{\mathrm{c,\,HER}}$ [1]	1			
I/C ratio	0.69	0.35	0.69	0.69
Pt wt.% in Pt/C	20	20	5	20
L_{Pt} (mg cm^{-2})	0.025	0.025	0.025	0.1
A_{Pt} (m^2 g^{-1})	100	100	150	100
$R_{\mathrm{K,\,HER}}$ (mOhm cm^2)	4.87	4.87	3.25	1.22
δ (µm)	10	10	40	40
$\rho_{\mathrm{H+,\,cath}}$ (Ohm cm) [3]	25	60	25	25
κ_{eff} (S cm^{-1})	0.04	0.017	0.04	0.04
s (cm^{-1})	2.27×10^3	3.51×10^3	1.39×10^3	2.27×10^3

Reference:

(1) Bernt, M.; Siebel, A.; Gasteiger, H. A. Analysis of Voltage Losses in PEM Water Electrolyzers with Low Platinum Group Metal Loadings. *J. Electrochem. Soc.* **2018**, *165* (5), F305–F314. https://doi.org/10.1149/2.0641805jes.

(2) Thompson, E. L.; Jorne, J.; Gu, W.; Gasteiger, H. A. PEM Fuel Cell Operation at −20°C. II. Ice Formation Dynamics, Current Distribution, and Voltage Losses within Electrodes. *J.*

Electrochem. Soc. **2008**, *155* (9), B887. https://doi.org/10.1149/1.2943203.

(3) Liu, Y.; Ji, C.; Gu, W.; Baker, D. R.; Jorne, J.; Gasteiger, H. A. Proton Conduction in PEM Fuel Cell Cathodes: Effects of Electrode Thickness and Ionomer Equivalent Weight. *J. Electrochem. Soc.* **2010**, *157* (8), B1154. https://doi.org/10.1149/1.3435323.
